# Appraisal of Gene-Environment Interactions in GWAS for Evidence-Based Precision Nutrition Implementation

**DOI:** 10.1007/s13668-022-00430-3

**Published:** 2022-08-11

**Authors:** Rodrigo San-Cristobal, Juan de Toro-Martín, Marie-Claude Vohl

**Affiliations:** 1 Centre Nutrition, Santé Et Société (NUTRISS), Institut Sur La Nutrition Et Les Aliments Fonctionnels (INAF), Université Laval, Québec, QC Canada; 2 School of Nutrition, Université Laval, Quebec, QC G1V 0A6 Canada

**Keywords:** Gene-environment interaction (GEI), Environmental factors, Genome-wide association study (GWAS), Precision nutrition, Metabolic syndrome, Cardiometabolic health

## Abstract

**Purpose of Review:**

This review aims to analyse the currently reported gene-environment (G × E) interactions in genome-wide association studies (GWAS), involving environmental factors such as lifestyle and dietary habits related to metabolic syndrome phenotypes. For this purpose, the present manuscript reviews the available GWAS registered on the GWAS Catalog reporting the interaction between environmental factors and metabolic syndrome traits.

**Recent Findings:**

Advances in omics-related analytical and computational approaches in recent years have led to a better understanding of the biological processes underlying these G × E interactions. A total of 42 GWAS were analysed, reporting over 300 loci interacting with environmental factors. Alcohol consumption, sleep time, smoking habit and physical activity were the most studied environmental factors with significant G × E interactions.

**Summary:**

The implementation of more comprehensive GWAS will provide a better understanding of the metabolic processes that determine individual responses to environmental exposures and their association with the development of chronic diseases such as obesity and the metabolic syndrome. This will facilitate the development of precision approaches for better prevention, management and treatment of these diseases.

**Supplementary Information:**

The online version contains supplementary material available at 10.1007/s13668-022-00430-3.

## Introduction

The transition to westernized lifestyles has stimulated an increase in food availability and the opening up of food choices across seasons and countries [1], leading an increase in food intake, and a reduction in physical activity which contributed to the development of the current obesity pandemic [2]. This burden of obesity, and mainly the increase in body fat, has been shown to be the trigger for inflammatory processes leading to the development of immuno-metabolic disorders [3]. Chronic accumulation of adipose tissue, mainly visceral fat, thus induces a chronic low-grade inflammatory state associated with insulin resistance, hyperlipidaemia and hypertension [4]. This constellation of metabolic disturbances forms the metabolic syndrome (MetS), which is associated with an increased risk of premature death.

The increase in the prevalence of MetS has turned into one of the major chronic non-communicable diseases impacting healthcare costs worldwide [5]. Currently, MetS criteria have different thresholds, varying according to specific characteristics of the target population, such as age [6], gender [7] or ancestry [8]. Despite these differences, MetS definitions shared some common features: overweight characterised by abdominal adiposity; impaired glucose tolerance; high blood pressure; decreased plasma high-density lipoprotein (HDL)-cholesterol; and increased triglyceride levels [9]. Regarding MetS prevention and treatment strategies, several studies have shown that individuals may respond differently to the same environmental factor [10, 11] or dietary exposure over the long term [12–14]. Similarly, recent results revealed that the main factors affecting postprandial glycaemic response are meal composition and genetic factors, while genetic variations did not significantly influence patients’ postprandial triglyceride levels [15••]. These findings reflect the growing assumption that one-size-fits-all nutritional recommendations are not optimal for everyone, highlighting the need for the development of precision nutrition approaches as a key step toward the effective prevention and treatment of MetS [16].

In this regard, genome-wide association studies (GWAS) aim to identify genetic markers associated with phenotypes by comparing the frequency of millions of genetic variants, such as the substitution of an individual base of the genome sequence, called single-nucleotide polymorphisms (SNPs), in a specific population with common ancestry [17]. The use of GWAS has increased with access to larger populations, such as the UK Biobank, the high-throughput sequencing and fine phenotyping [18]. Studies analysing gene–gene and gene-environment (G × E) interactions have increased in recent years, in parallel with GWAS. These studies aim to elucidate the network of interactions involved in the development of complex diseases, such as obesity and MetS, in which multiple genes and environmental factors may modulate the individual risk for disease development [19].

The increasing availability of larger population samples and new methodologies for modelling interactions in complex diseases is allowing researchers to integrate and combine datasets of different natures. These new multivariate models shed light on disease complexity and enable the development of new tools for precision medicine and nutrition approaches [20, 21]. In this respect, nutrigenetic and nutrigenomic studies are focused on highlighting the key role of G × E interactions involving dietary habits and lifestyle factors in MetS [22, 23]. It should be noted that this type of research has shown that, despite the impact of genetic predisposition to the disease, this susceptibility can be mitigated. Environmental factors can aggravate or mitigate the effects of genetic factors. One can think simply of nutritional approaches in which a food or nutrient can be avoided or replaced so as not to exacerbate the effect of genetic factors on health, or supplemented if the effect of the mutation is to limit its availability [14]. In this regard, one of the most described examples is the presence of polymorphisms related with the disruption of one-carbon metabolism associated with the development of metabolic syndrome traits and the role of B vitamin supplementation as methyl donors for DNA methylation and its implications in cardiometabolic health and offspring well-being [24]. Despite these advances in recent years, several authors have emphasised the need for further nutrigenetic studies to strengthen the evidence on these complex relationships between lifestyle and genetics, and to apply more effective tools for precision nutrition counselling [25, 26]. In order to develop new intervention strategies and to implement new standardized procedures of precision nutrition applications, these novel studies need to be supported and integrate previous evidence [27, 28].

With this in mind, the present review aims to analyse the current evidence on G × E interactions related to MetS and reported in the GWAS Catalog, concretely on environmental factors related mainly to lifestyle and dietary habits.

## Search Strategy and Selection Criteria

The cumulative knowledge from GWAS has paved the way for the study of complex traits by considering pleiotropic effects between genetic variants for multiple complex traits [29]. As the number of published GWAS increased [30], it became necessary to systematically compile the information provided by these studies. To this end, the GWAS Catalog (available at https://www.ebi.ac.uk/gwas/) has been collecting information from GWAS since 2005 and provides a public database that summarizes the compiled information associated with a large number of traits, such as obesity, diabetes, cardiovascular disease or different types of cancer [31]. Over the years, the GWAS Catalog has also evolved to adapt to new findings and strategies in the field and has incorporated new data including large meta-analysis, Mendelian randomization studies and evidence related to interactions [32]. For this reason, the studies included in this review were limited to GWAS registered in the GWAS Catalog [31].

**Fig. 1:**
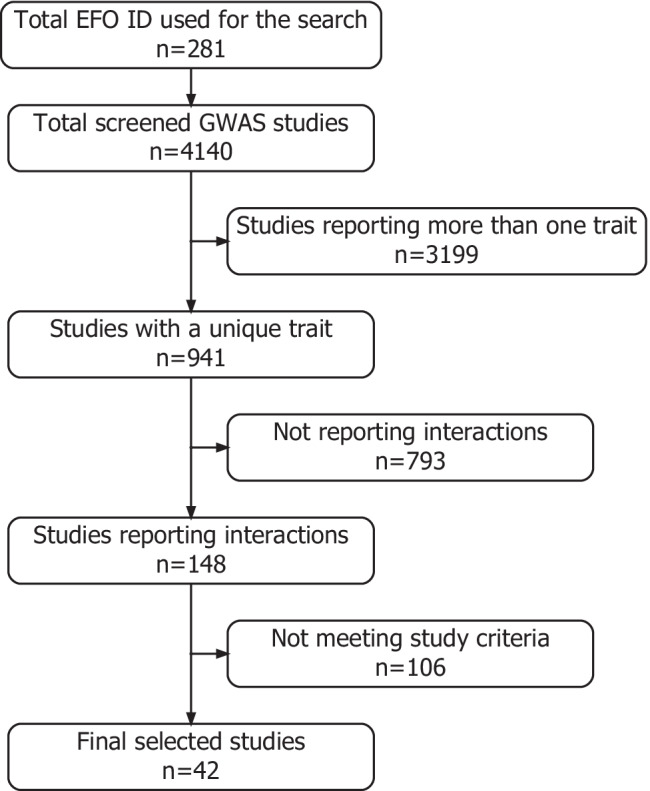
Flowchart of the GWAS study selection

The present review aims to systematically search, identify and provide a narrative synthesis of the GWAS that assessed interactions between genetic variants and environmental factor with impact on related metabolic syndrome traits. Data were searched using the *gwasrapid* R package [33] to query the registered studies for the following MetS traits: obesity, glucose metabolism, cholesterol, blood pressure and triglycerides, as shown in Supplementary Table 1. The search was conducted between March 20 and April 14, 2022. A total of 281 Experimental Factor Ontology (EFO) were used to conduct the inquiry on the GWAS Catalog database (Fig. 1). From these selected traits, only GWAS reporting significant interactions with at least one of the following environmental factors: dietary intake, physical activity, smoking and sleep habits, were selected to be included in the review. Thus, out of a total of 941 GWAS (without duplication of numbers for studies involving more than one trait), only 148 reported a G × E interaction and 42 reported interactions with the environmental factors studied. The selected articles were published between 2011 and 2021.

The information collected from the GWAS Catalog included author(s), journal and year of publication, size and ethnicity of the cohort population, information from the reported interaction, SNP and the nearest mapped gene, associated trait and the environmental factor interacting with the SNP, as well as covariates included in the model (Supplementary Tables 2 to 6).

A complementary pathway enrichment analysis was performed with genes having at least one SNP showing a significant G × E interaction within the retained studies. The functional significance of these genes was explored by using the *clusterProfiler* R package [34] and the Gene Ontology database [35]. Pathways were considered significantly enriched at FDR-adjusted *p* value < 0.05.

## Environmental Factors Interacting in GWAS

**Fig. 2:**
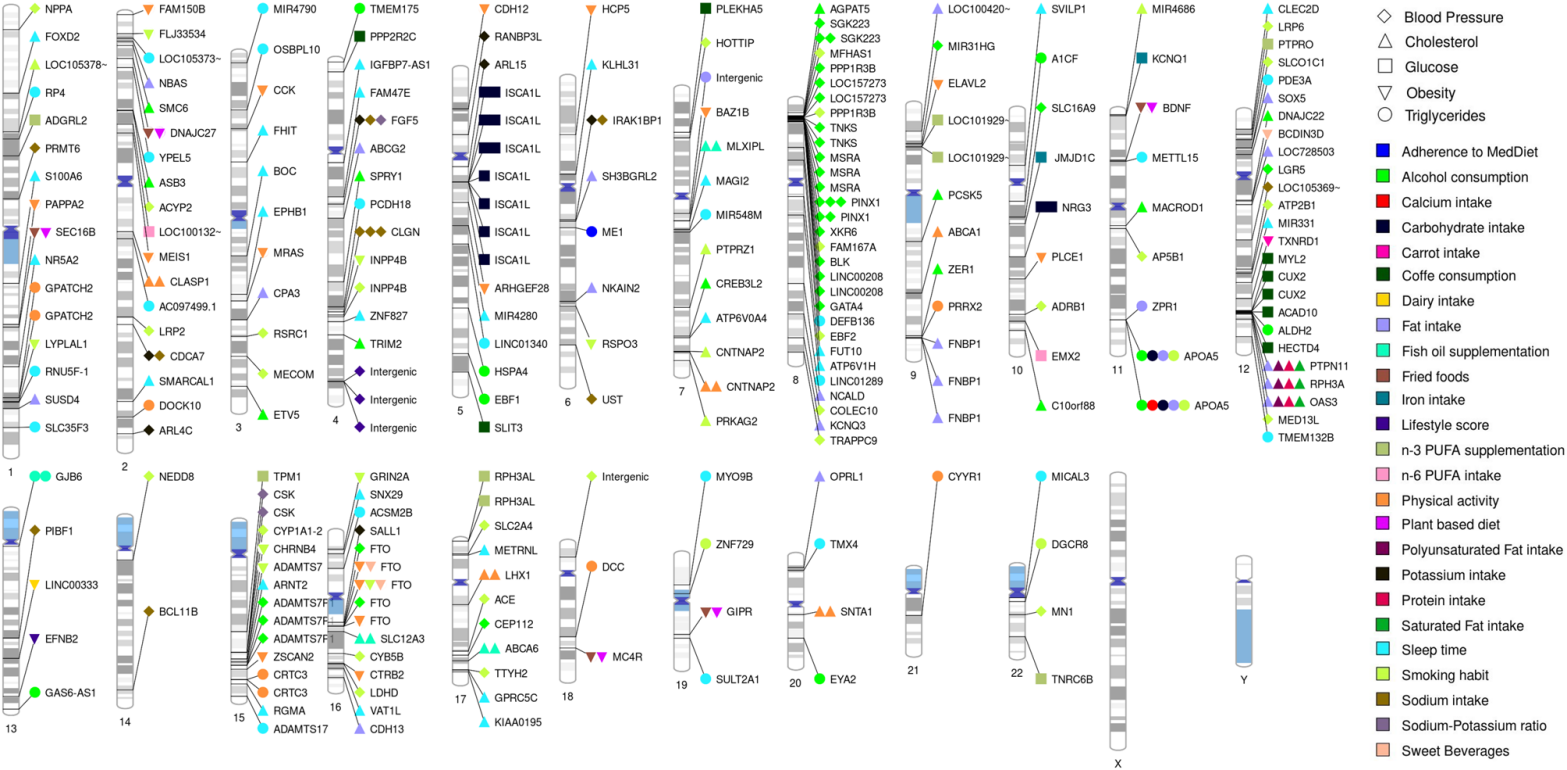
Summary of genes and nearby genes exhibiting gene-environment interactions with environmental factors in the GWAS Catalog. The figure summarizes all the mapped genes and nearby genes to the reported SNPs in the GWAS Catalog showing significant gene-environment interactions. The figure was built using PhenoGram [38]

**Fig. 3:**
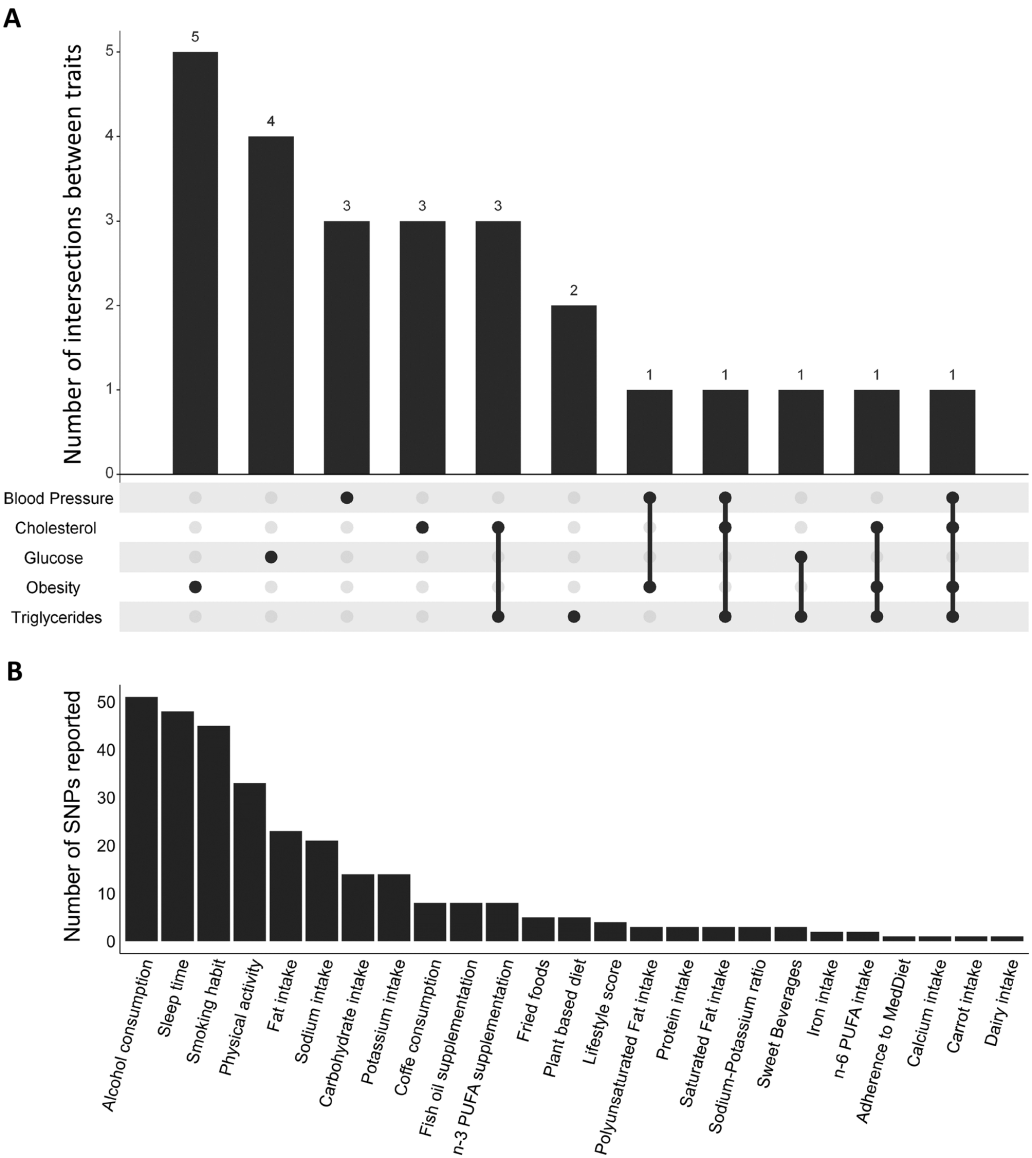
Summary of the G × E interactions reported in the present review. Panel **A** shows an upset plot of the intersection between the 25 environmental factors reported in the GWAS Catalog and the metabolic syndrome traits presenting significant gene-environment interactions. Panel **B** shows a density plot of the frequency of SNPs reporting significant interactions with environmental factors

A total of 310 interactions with 25 environmental factors reporting significant interactions with MetS traits were identified (Fig. 2). The four most common environmental factors were alcohol consumption with 51 SNPs, sleep time with 48 SNPs, smoking habit with 45 SNPs and physical activity with 33 SNPs (Fig. 3B). These factors were grouped into four main clusters: dietary habits, physical activity, smoking and sleeping habits. Amongst these groups, “dietary habits” was the group with the greatest variety of factors, including adherence to the Mediterranean diet, alcohol consumption, calcium intake, carbohydrate intake, carrot consumption, coffee consumption, dairy intake, fat intake, fish oil supplementation, fried food consumption, iron intake, n-3 polyunsaturated fatty acid (PUFA) supplementation, n-6 PUFA intake, total PUFA intake, plant-based diet, potassium intake, protein intake, saturated fat intake, sodium intake and sweetened beverages (Fig. 3B). However, although common dietary habits are known to have a direct impact on weight or health maintenance, such as coffee consumption, fish oil supplementation or the consumption of fried foods or sweet beverages, most GWAS investigating these dietary traits reported interactions with less than 10 SNPs, highlighting the need for further and larger studies [36]. Furthermore, although several studies have investigated the interactions between G × E and these environmental factors, most of these are candidate gene-based studies and therefore, only a limited number of genes and polymorphisms have been studied. In this respect, the development of new GWAS including G × E interactions could provide more eloquent evidence to improve the predictive power of genetic markers from these studies [37]. In addition, different technologies were used to analyse the genome in the revised articles. Affymetrix and Illumina platforms represent almost the 95% of the articles reviewed (55% and 39% respectively).

On the other hand, we found that 17 out of the 25 reported environmental factors were associated with only one MetS trait, and only three of them (smoking habit, physical activity and alcohol consumption) were associated with more than three MetS traits (Fig. 3A). To our knowledge, the development of models combining MetS traits associated with the same environmental factor may shed light on the pleiotropic interactions that occur during the development of immuno-metabolic alterations [39, 40]. In order words, the development of new models combining several MetS traits with environmental factors is still needed in the field to reveal hidden layers of complexity. Ultimately, this will facilitate the development of more effective nutritional strategies for the prevention and management of complex, multifactorial diseases such as MetS. This also emphasises the need to develop further GWAS models. In the present review, we found that a total of 12 GWAS considered joint MetS traits, but none of them considered interactions with environmental factors in their analyses.

## MetS Traits Interacting with Environmental Factors

### Obesity

Obesity is one of the most studied metabolic disorders because of its increasing prevalence in recent decades and its impact on life quality and overall health. Similarly, obesity has been the study subject of several GWAS aimed at shedding light on the polygenic architecture of the disease [41]. At the same time, external modulators such as environmental factors, cultural values, food choices, economic factors, education or stress add complexity to the integration of interindividual differences in obesity and MetS management and prevention programs [42].

In addition, some authors suggest that the difficulty in unravelling risk factors explaining the interindividual variability observed in obesity and MetS is related to the complex interactions with environmental factors [43, 44]. Despite this, our search in the GWAS Catalog revealed only eight studies on obesity considering G × E interactions (Supplementary Table 2). In this regard, *FTO* is one of the most representative obesity-related genes, which has been widely studied due to its association with multiple obesity phenotypes and diabetes [45]. Additionally, *FTO* exhibits pleiotropic effects with BMI-independent traits [46], with interactions with other genes [47] and with environmental factors, such as diet [14]. Herein, we found that *FTO* was the only gene associated with two different traits: obesity [48••, 49] and blood pressure [50], and interacting with four different environmental factors: physical activity [48••, 49], smoking habit [48••], sweet beverage [48••] and alcohol consumption [50] (Supplementary Tables 2 and 4). The influence of physical activity on the obesogenic effect of SNPs located at *FTO* locus has been widely studied, displaying a consistent attenuation. A meta-analysis reported that active individuals exhibited around 20% reduction of the BMI-increasing effects of *FTO*-associated SNPs [49], supporting previous results reporting 30% attenuation of the genetic effect [51, 52]. However, the mechanism underlying this gene-physical activity interaction remains to be elucidated due to the multiple regulatory processes and shared pathways [53, 54]. In addition, the interaction network is complex, with many G × E interactions with *FTO* [14].

In this respect, increased adiposity has been linked to increased blood pressure by Mendelian randomisation using SNPs located at the FTO and MC4R loci as instrumental variants [55]. In fact, the use of genetic factors of obesity as an instrumental variable provides a valuable tool to understand the biological mechanism linking obesity to metabolic complications. A recent review suggests that the application of information from obesity-related GWAS data can help shed light on the etiological mechanism of obesity-related metabolic risk [56]. Furthermore, a recent study carried out on twins showed that environmental factors such as smoking status, alcohol consumption and physical activity are key triggers in the development of obesity-associated hypertension [57], and a previous meta-analysis suggested that this association may be mediated by a SNP at the *FTO* locus [58]. Similarly, another GWAS also showed evidence linking alcohol intake with blood pressure through genes previously associated with alcohol intake, such as *PINX1*, *GATA4*, *BLK*, *FTO* and *GABBR2* [50]. Additionally, *ACE*, *ADRB1* and *CSK*, genes interacting with smoking exposure and 24-h urinary sodium/potassium ratio (Supplementary Table 4), were also reported to interact with hypotensive drugs [59]. These results suggest that some of these G × E interactions may share the same underlying mechanism as for the effects observed with environmental factors themselves. Thus, meta-analysis [39] and Mendelian randomization studies [60] have analysed the complexity of the pleiotropic effects on cardiovascular disease variants, revealing the presence of several variants exhibiting multiple associations with obesity and metabolic traits. These results provided additional evidence to explain the classical links between metabolic alterations as cardiovascular risk factor and its heritance.

### Triglyceride

The *APOA5* gene exhibits one of the strongest effects on plasma triglyceride levels [61] and is also implicated in the development of obesity and MetS [62], interacting with poor lifestyle factors [63]. Several SNPs located within this locus have been associated with a deficient function of the protein associated with severe hypertriglyceridemia [61]. However, *APOA5* has also been associated with moderate hypertriglyceridemia, related with insulin resistance and increased risk of atherogenic dyslipidaemia [64]. In addition, some studies have suggested that the impact of *APOA5* on plasma triglyceride levels is enhanced by the increase of adiposity [65]. Likewise, *APOA5* effects on plasma triglyceride levels seem to be mediated by its interaction with multiple environmental factors, such as smoking habit, carbohydrate, fat, alcohol and calcium intake (Supplementary Table 6) [66]. Some authors have suggested that the interaction between postprandial increase of plasma triglyceride levels and environmental factors may be mediated by epigenetic factors, contributing to the modulation of the risk for cardiovascular disease [67]. More specifically, the study carried out by Wojczynski et al. [68] described a significant association between five different methylation marks and a SNP within *ZPR1*, a gene located close to the *APOA1/C3/A4/A5* cluster, and showing a significant interaction between postprandial plasma triglyceride levels and a high-fat meal (Supplementary Table 6). A subsequent study also found a significant association between methylation marks within *APOA5* with 20 SNPs in the nearby region, and significantly associated with the postprandial plasma triglyceride response after a high-fat meal [69]. These results suggest that the interindividual differences attributable to lipid-related SNPs may be partly explained by variations in epigenetic marks caused by environmental factors. At the same time, these results emphasise the need for further studies that integrate epigenetic analyses and GWAS to provide a better understanding of the biological processes behind these interactions [70].

### Cholesterol

Similarly to *APOA5*, *CNTNAP2* seems to interact with physical activity [71] and smoking habit [72] to modulate plasma cholesterol levels. Despite this gene has been widely associated with multiple neurodevelopmental disorders [73], it has also been linked with energy homeostasis and body weight regulation [74]. A recent study combining GWAS and transcriptomics has found a SNP within *CNTNAP2* locus associated with plasma ghrelin levels [75•]. Furthermore, two different epigenetic studies described a decrease of methylation levels in this gene associated with the smoking habits of the mother and lower birth weight of the offspring [76, 77]. Another recent study analysed the long-term effects of smoking habits of the mother on methylation levels of their offspring during adolescence and its association with the cardiometabolic risk factors [78]. They found differential methylation marks in *FTO*, *CYP1A1* and *CNTNAP2* genes, amongst others. In addition, methylation levels of *FTO* and *CYP1A1* genes were also associated with blood pressure, plasma triglyceride levels and HDL-cholesterol levels. Herein, *FTO* was identified as being part of the “brown fat cell differentiation” pathway (Supplementary Fig. 1A), significantly enriched in the present study. Similarly, the abovementioned *ZPR1* gene was also identified within the “axon development” pathway (Supplementary Fig. 1A), as well as *BDNF*, a gene closely associated with the metabolic regulation of body weight, along with *FTO* [41]. In this regard, we found a total of 14 metabolic pathways to be significantly enriched with genes having at least one SNP showing a significant G × E interaction (Supplementary Fig. 1B). In addition to those already mentioned, the most relevant pathways were related to cardiovascular health, and more concretely, to blood pressure regulation (Supplementary Fig. 1B), with a significant presence of the aforementioned *ACE* and *ADRB1* genes in these pathways*,* suggesting an important role of G × E interactions in controlling this metabolic trait.

### Blood Pressure

Blood pressure was in fact the most commonly studied trait in the GWAS Catalog with 15 studies and 78 different genes showing G × E interactions with environmental factors (Supplementary Table 4). In contrast, we identified only five studies reporting G × E interactions related to glucose levels (Supplementary Table 3). Interestingly, three of these studies computed a genetic risk score (GRS) to collect the genetic structure of the individuals and its interaction with environmental factors [79, 80]. GRS constructed with GWAS information incorporates the effects of multiple SNPs across the genome and captures the interindividual variability. This information then allows the stratification of individuals according to their risk to exhibit plasma glucose alterations. In this sense, the risk prediction of a wide range of SNPs related to glucose homeostasis appears to be dependent of clinical features, such as BMI [81]. Similarly, G × E interactions collected in this review (Supplementary Table 2) may provide a valuable tool for the management of glucose metabolism disorders through the application of precision nutrition advice. The integration of methylation information with GWAS in the risk assessment of complex diseases may further enable a more comprehensive interpretation of the biological processes involved [82•].

## Perspectives of the GWAS with Environmental Interactions

As seen above, one of the most popular applications of GWAS is the estimation of GRS, also known as polygenic risk scores (PRS), based on the addition of multiple small effects across SNPs associated with a specific trait and able to capture part of the individual susceptibility to develop a disease. These PRS aim to capture the contribution of heredity to multifactorial complex diseases by estimating a risk score based on multiple genetic variations that reflects the risk of developing a disease compared to a population with a common genetic sequence without these genetic variations [83]. This has been proposed as a powerful public health tool for the prevention and screening of the population to detect high-risk groups [84]. In this regard, other studies suggest that the risk prediction of PRS may have a modest enhancement added to the traditional guideline-recommended clinical risk factors [85, 86]. However, these studies computed PRS in the traditional way, selecting the SNPs to be included based on their association with the trait and weighted by the degree of association, but without considering the weight of potential interactions with environmental factors [87••]. The development of new strategies and more efficient statistical analyses considering G × E interactions in the assessment of complex diseases are needed in the field [88, 89]. In addition, the inclusion of G × E interactions in the construction of PRS is expected to be the main key for improving the predictive power of precision medicine and nutrition tools [87••, 90]. For this reason, the construction of new PRS accounting for G × E interactions that may provide improvements in performance prediction and risk stratification has been proposed [83].

**Fig. 4:**
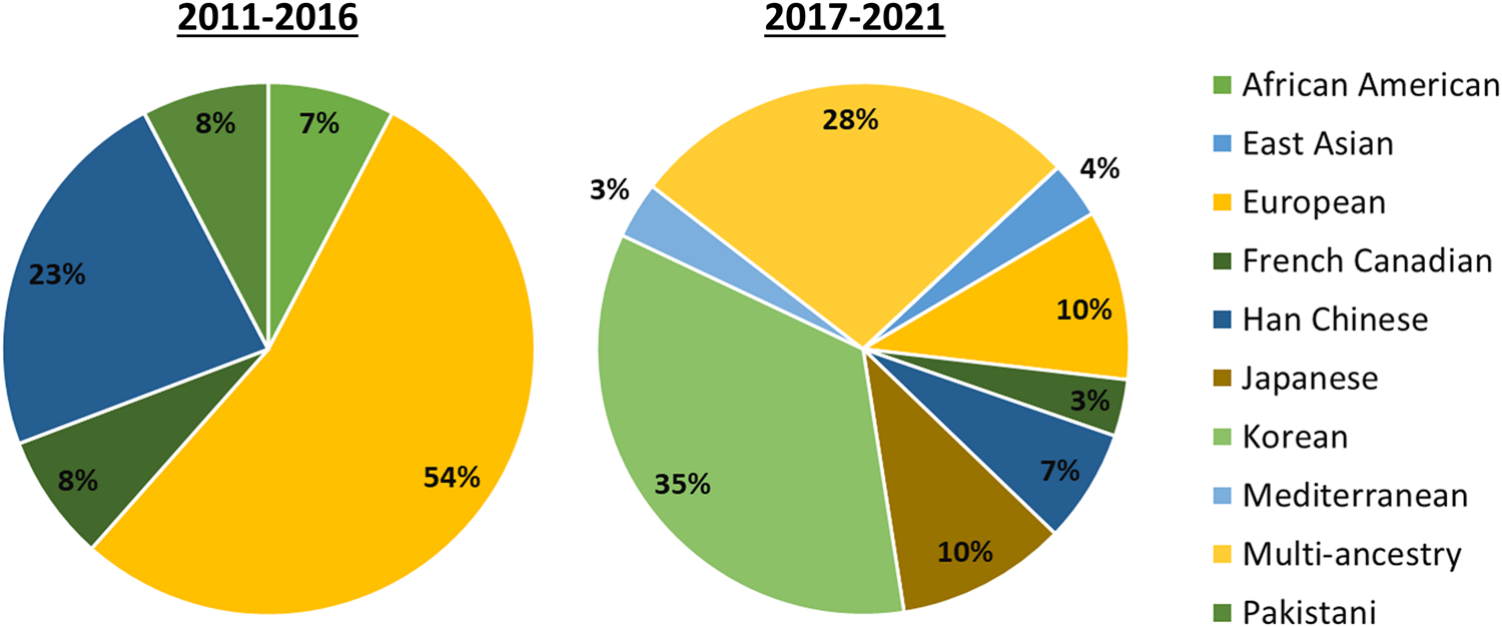
Frequency of population ancestry in the selected GWAS over two time periods: 2011 to 2016 (*n* = 13) and 2017 to 2021 (*n* = 29)

Despite these promising results, some authors are still conservative with the application of PRS because of the limited generalizability of GWAS results and the insufficient diversity of studied populations [91]. This is due, amongst other things, to the fact that several studies have been conducted on populations of European origin [48••, 68, 92–100] and to difference in sample size between studies (from 138 to 347,158 individuals) [48••, 101]. Thus, this may create a significant bias for risk prediction when these results are extrapolated to other populations [102]. This is also noticeable in the results of this review, as only seven of the studies conducted analyses in a multi-ethnic population (Supplementary Tables 2 to 5). However, an increase in the number of GWAS incorporating populations of different ancestry has been observed over the past years. Accordingly, we observed a reduction in the percentage of GWAS based on European populations, from 54% during 2011–2016 to 10% during 2017–2021 (Fig. 4). Interestingly, the number of GWAS carried out in Korean populations has also raised during the 2017–2021 period (35%), as compared to 2011–2016 (7%) (Fig. 4). Increasing the number of ancestries in these studies will help to reduce inequalities and provide a truly comprehensive picture of the genetic architecture of human diseases [103]. However, there is still an urgent need to increase the sample size of these underrepresented populations; the expectations are hopeful due to the increased availability of large biobanks and cohorts that will increase the number and the diversity of studied populations [30].

## Conclusion

Environmental and genetic factors contribute to the susceptibility of chronic complex diseases, such as MetS. The improvement of analytical and computational approaches in the past years has provided tones of information leading to a better understanding of the biological processes underlying the development of these diseases [104, 105]. However, the integration of information from different natures is needed for a full comprehension of the interplay between genetic and environmental factors. This review updates the previous efforts to compile G × E interactions found in GWAS [106]. The implementation of GWAS including G × E interactions is a great opportunity for the development of more effective approaches for the prevention and management of these disorders in the clinical practice. The findings shown in the present review suggest that there are still some challenges that need to be overcome. Advances in data harmonisation, the integration of multi-omic approaches and the use of larger and multi-ancestry populations, as well as the control for environmental exposures, will lead to more comprehensive GWAS. This will contribute to the implementation of accurate precision nutrition approaches through the most appropriate dietary and lifestyle advice for each individual.

## Supplementary Information

Below is the link to the electronic supplementary material.

Supplementary file1 (PDF 584 KB)
