# Supplementary material for: Appraisal of Gene-Environment Interactions in GWAS for Evidence-Based Precision Nutrition Implementation
Source: Curr Nutr Rep. 2022 Aug 11;11(4):563–73. doi: 10.1007/s13668-022-00430-3 (PMC9750926; doi:10.1007/s13668-022-00430-3)
Supplement: Supplementary file 1 — Supplementary file1 (PDF 584 KB) [file 13668_2022_430_MOESM1_ESM.pdf]

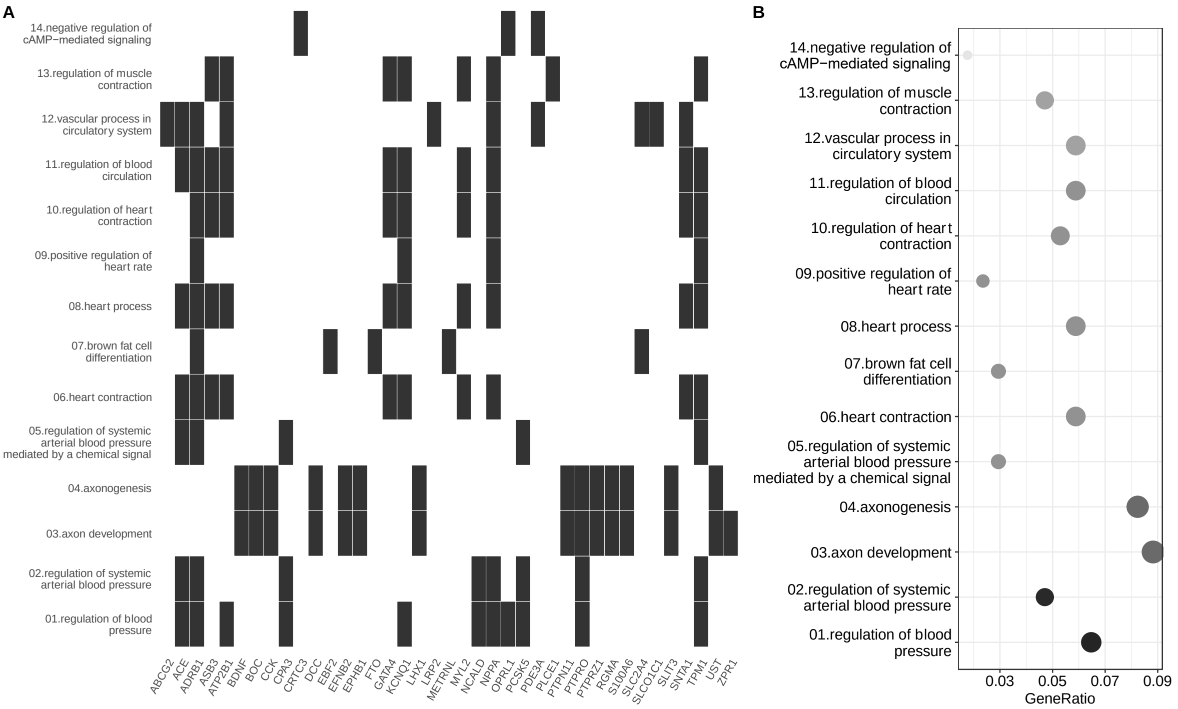

Supplementary Figure 1: Functional pathways significantly enriched with genes reported in the GWAS Catalog. Panel A shows a heatplot with top Gene Ontology-Biological Process (GO-BP) terms significantly enriched with genes showing at least one SNP showing a significant gene-environment interaction in the GWAS Catalog. Panel B shows a dotplot with top GO-BP terms significantly enriched. Each dot has a particular size and tone that represent the number of genes in the pathway and the FDR-adjusted p-value, respectively. GeneRatio represents the number of genes present in a given pathway relative to the size of the gene list for that pathway. Pathways were considered significantly enriched at FDR-adjusted p-value < 0.05.

Supplementary Table 1: Traits included in the GWAS Catalog search.

Efo ID	Trait	url
**HP_0011015**	abnormal blood glucose concentration	http://purl.obolibrary.org/obo/HP_0011015
**HP_0003119**	abnormal circulating lipid concentration	http://purl.obolibrary.org/obo/HP_0003119
**HP_0011014**	abnormal glucose homeostasis	http://purl.obolibrary.org/obo/HP_0011014
**EFO_0002546**	abnormal glucose tolerance	http://www.ebi.ac.uk/efo/EFO_0002546
**HP_0030972**	abnormal systemic blood pressure	http://purl.obolibrary.org/obo/HP_0030972
**EFO_0006831**	acute insulin response measurement	http://www.ebi.ac.uk/efo/EFO_0006831
**EFO_0004764**	adipose tissue measurement	http://www.ebi.ac.uk/efo/EFO_0004764
**MONDO_0002046**	alcohol abuse	http://purl.obolibrary.org/obo/MONDO_0002046
**EFO_0004776**	alcohol and nicotine codependence	http://www.ebi.ac.uk/efo/EFO_0004776
**EFO_0007878**	alcohol consumption measurement	http://www.ebi.ac.uk/efo/EFO_0007878
**EFO_0008017**	alcohol dehydrogenase [NADP+] measurement	http://www.ebi.ac.uk/efo/EFO_0008017
**MONDO_0007079**	alcohol dependence	http://purl.obolibrary.org/obo/MONDO_0007079
**EFO_0007835**	alcohol dependence measurement	http://www.ebi.ac.uk/efo/EFO_0007835
**EFO_0004329**	alcohol drinking	http://www.ebi.ac.uk/efo/EFO_0004329
**EFO_0009113**	alcohol exposure measurement	http://www.ebi.ac.uk/efo/EFO_0009113
**EFO_0009458**	alcohol use disorder measurement	http://www.ebi.ac.uk/efo/EFO_0009458
**EFO_0004777**	alcohol withdrawal	http://www.ebi.ac.uk/efo/EFO_0004777
**EFO_1000800**	alcohol withdrawal delirium	http://www.ebi.ac.uk/efo/EFO_1000800
**EFO_1000802**	alcoholic liver cirrhosis	http://www.ebi.ac.uk/efo/EFO_1000802
**EFO_0008573**	alcoholic liver disease	http://www.ebi.ac.uk/efo/EFO_0008573
**EFO_1002013**	alcoholic pancreatitis	http://www.ebi.ac.uk/efo/EFO_1002013
**MONDO_0021698**	alcohol-related disorders	http://purl.obolibrary.org/obo/MONDO_0021698
**EFO_0004302**	anthropometric measurement	http://www.ebi.ac.uk/efo/EFO_0004302
**EFO_0005200**	antiphospholipid antibody measurement	http://www.ebi.ac.uk/efo/EFO_0005200
**EFO_0002689**	antiphospholipid syndrome	http://www.ebi.ac.uk/efo/EFO_0002689
**EFO_0010096**	artificially sweetened beverage consumption measurement	http://www.ebi.ac.uk/efo/EFO_0010096

**EFO_0007777**	base metabolic rate measurement	http://www.ebi.ac.uk/efo/EFO_0007777
**EFO_0010092**	bitter alcoholic beverage consumption measurement	http://www.ebi.ac.uk/efo/EFO_0010092
**EFO_0010089**	bitter beverage consumption measurement	http://www.ebi.ac.uk/efo/EFO_0010089
**EFO_0010093**	bitter non-alcoholic beverage consumption measurement	http://www.ebi.ac.uk/efo/EFO_0010093
**EFO_0004325**	blood pressure	http://www.ebi.ac.uk/efo/EFO_0004325
**EFO_0006943**	blood pressure change measurement	http://www.ebi.ac.uk/efo/EFO_0006943
**EFO_0007737**	BMI-adjusted adiponectin measurement	http://www.ebi.ac.uk/efo/EFO_0007737
**EFO_0008036**	BMI-adjusted fasting blood glucose measurement	http://www.ebi.ac.uk/efo/EFO_0008036
**EFO_0008037**	BMI-adjusted fasting blood insulin measurement	http://www.ebi.ac.uk/efo/EFO_0008037
**EFO_0008038**	BMI-adjusted hip bone size	http://www.ebi.ac.uk/efo/EFO_0008038
**EFO_0008039**	BMI-adjusted hip circumference	http://www.ebi.ac.uk/efo/EFO_0008039
**EFO_0007793**	BMI-adjusted leptin measurement	http://www.ebi.ac.uk/efo/EFO_0007793
**EFO_0011044**	BMI-adjusted neck circumference	http://www.ebi.ac.uk/efo/EFO_0011044
**EFO_0007789**	BMI-adjusted waist circumference	http://www.ebi.ac.uk/efo/EFO_0007789
**EFO_0007788**	BMI-adjusted waist-hip ratio	http://www.ebi.ac.uk/efo/EFO_0007788
**EFO_0005106**	body composition measurement	http://www.ebi.ac.uk/efo/EFO_0005106
**EFO_0004341**	body fat distribution	http://www.ebi.ac.uk/efo/EFO_0004341
**EFO_0007800**	body fat percentage	http://www.ebi.ac.uk/efo/EFO_0007800
**EFO_0004340**	body mass index	http://www.ebi.ac.uk/efo/EFO_0004340
**EFO_0004338**	body weight	http://www.ebi.ac.uk/efo/EFO_0004338
**EFO_0004566**	body weight gain	http://www.ebi.ac.uk/efo/EFO_0004566
**EFO_0005245**	body weight loss	http://www.ebi.ac.uk/efo/EFO_0005245
**EFO_0004324**	body weights and measures	http://www.ebi.ac.uk/efo/EFO_0004324
**EFO_0006521**	calcium intake measurement	http://www.ebi.ac.uk/efo/EFO_0006521
**EFO_0010811**	carbohydrate intake measurement	http://www.ebi.ac.uk/efo/EFO_0010811
**EFO_0007723**	carotid artery mean blood pressure measurement	http://www.ebi.ac.uk/efo/EFO_0007723
**EFO_0005942**	chemotherapy-induced hypertension	http://www.ebi.ac.uk/efo/EFO_0005942
**EFO_0009132**	cholesterol efflux capacity measurement	http://www.ebi.ac.uk/efo/EFO_0009132
**EFO_0006824**	cis/trans-18:2 fatty acid measurement	http://www.ebi.ac.uk/efo/EFO_0006824

**EFO_0020897**	citrus intake measurement	http://www.ebi.ac.uk/efo/EFO_0020897
**EFO_0004330**	coffee consumption	http://www.ebi.ac.uk/efo/EFO_0004330
**EFO_0006781**	coffee consumption measurement	http://www.ebi.ac.uk/efo/EFO_0006781
**EFO_0000400**	diabetes mellitus	http://www.ebi.ac.uk/efo/EFO_0000400
**EFO_0006842**	diabetes mellitus biomarker	http://www.ebi.ac.uk/efo/EFO_0006842
**EFO_0004596**	diabetes mellitus type 2 associated cataract	http://www.ebi.ac.uk/efo/EFO_0004596
**EFO_0006336**	diastolic blood pressure	http://www.ebi.ac.uk/efo/EFO_0006336
**EFO_0006945**	diastolic blood pressure change measurement	http://www.ebi.ac.uk/efo/EFO_0006945
**EFO_0008111**	diet measurement	http://www.ebi.ac.uk/efo/EFO_0008111
**EFO_0010816**	dietary fat liking measurement	http://www.ebi.ac.uk/efo/EFO_0010816
**EFO_0008355**	dietary heme iron intake measurement	http://www.ebi.ac.uk/efo/EFO_0008355
**EFO_0008470**	dietary potassium intake measurement	http://www.ebi.ac.uk/efo/EFO_0008470
**EFO_0004315**	drinking behavior	http://www.ebi.ac.uk/efo/EFO_0004315
**EFO_0004772**	early onset hypertension	http://www.ebi.ac.uk/efo/EFO_0004772
**EFO_0005109**	energy expenditure	http://www.ebi.ac.uk/efo/EFO_0005109
**EFO_0008005**	energy expenditure measurement	http://www.ebi.ac.uk/efo/EFO_0008005
**EFO_0003939**	energy intake	http://www.ebi.ac.uk/efo/EFO_0003939
**EFO_0009374**	energy intake measurement	http://www.ebi.ac.uk/efo/EFO_0009374
**MONDO_0001134**	essential hypertension	http://purl.obolibrary.org/obo/MONDO_0001134
**EFO_0020904**	esterified cholesterol change measurement	http://www.ebi.ac.uk/efo/EFO_0020904
**EFO_0008589**	esterified cholesterol measurement	http://www.ebi.ac.uk/efo/EFO_0008589
**EFO_0007875**	excessive daytime sleepiness measurement	http://www.ebi.ac.uk/efo/EFO_0007875
**EFO_0000483**	exercise	http://www.ebi.ac.uk/efo/EFO_0000483
**HP_0410139**	Exercise induced anaphylaxis	http://purl.obolibrary.org/obo/HP_0410139
**EFO_0004328**	exercise test	http://www.ebi.ac.uk/efo/EFO_0004328
**EFO_0004911**	familial hypercholesterolemia	http://www.ebi.ac.uk/efo/EFO_0004911
**EFO_0010120**	fasting blood glucose change measurement	http://www.ebi.ac.uk/efo/EFO_0010120
**EFO_0004465**	fasting blood glucose measurement	http://www.ebi.ac.uk/efo/EFO_0004465
**EFO_0004466**	fasting blood insulin measurement	http://www.ebi.ac.uk/efo/EFO_0004466

**EFO_0005409**	fat body mass	http://www.ebi.ac.uk/efo/EFO_0005409
**EFO_0010809**	fat intake measurement	http://www.ebi.ac.uk/efo/EFO_0010809
**EFO_0020875**	fatty acid change measurement	http://www.ebi.ac.uk/efo/EFO_0020875
**EFO_0009791**	fatty acid desaturase enzyme activity measurement	http://www.ebi.ac.uk/efo/EFO_0009791
**EFO_0005110**	fatty acid measurement	http://www.ebi.ac.uk/efo/EFO_0005110
**EFO_0010914**	fatty acid-binding protein, adipocyte measurement	http://www.ebi.ac.uk/efo/EFO_0010914
**EFO_0020372**	fatty acid-binding protein, epidermal measurement	http://www.ebi.ac.uk/efo/EFO_0020372
**EFO_0020373**	fatty acid-binding protein, heart measurement	http://www.ebi.ac.uk/efo/EFO_0020373
**EFO_0010139**	fish consumption measurement	http://www.ebi.ac.uk/efo/EFO_0010139
**EFO_0600007**	fish oil supplement exposure measurement	http://www.ebi.ac.uk/efo/EFO_0600007
**EFO_0020905**	free cholesterol change measurement	http://www.ebi.ac.uk/efo/EFO_0020905
**EFO_0008591**	free cholesterol measurement	http://www.ebi.ac.uk/efo/EFO_0008591
**EFO_0009698**	gestational blood glucose measurement	http://www.ebi.ac.uk/efo/EFO_0009698
**EFO_0008531**	GLP-1-stimulated insulin response	http://www.ebi.ac.uk/efo/EFO_0008531
**EFO_0020881**	glucose change measurement	http://www.ebi.ac.uk/efo/EFO_0020881
**EFO_0006833**	glucose effectiveness measurement	http://www.ebi.ac.uk/efo/EFO_0006833
**EFO_0006896**	glucose homeostasis measurement	http://www.ebi.ac.uk/efo/EFO_0006896
**EFO_0004468**	glucose measurement	http://www.ebi.ac.uk/efo/EFO_0004468
**EFO_0009392**	glucose metabolism decline measurement	http://www.ebi.ac.uk/efo/EFO_0009392
**EFO_0009367**	glucose metabolism measurement	http://www.ebi.ac.uk/efo/EFO_0009367
**EFO_0004307**	glucose tolerance test	http://www.ebi.ac.uk/efo/EFO_0004307
**EFO_0010484**	glucose-1-phosphate measurement	http://www.ebi.ac.uk/efo/EFO_0010484
**EFO_0020407**	glucose-6-phosphate isomerase measurement	http://www.ebi.ac.uk/efo/EFO_0020407
**EFO_0010485**	glucose-6-phosphate measurement	http://www.ebi.ac.uk/efo/EFO_0010485
**EFO_0008464**	glucose-dependent insulinotropic peptide measurement	http://www.ebi.ac.uk/efo/EFO_0008464
**EFO_0020869**	glycerophospholipid change measurement	http://www.ebi.ac.uk/efo/EFO_0020869
**EFO_0007630**	glycerophospholipid measurement	http://www.ebi.ac.uk/efo/EFO_0007630
**EFO_0010094**	grapefruit juice consumption measurement	http://www.ebi.ac.uk/efo/EFO_0010094
**EFO_0007805**	HDL cholesterol change measurement	http://www.ebi.ac.uk/efo/EFO_0007805

**EFO_0009184**	heart rate response to exercise	http://www.ebi.ac.uk/efo/EFO_0009184
**EFO_0009185**	heart rate response to recovery post exercise	http://www.ebi.ac.uk/efo/EFO_0009185
**EFO_0005851**	height-adjusted body mass index	http://www.ebi.ac.uk/efo/EFO_0005851
**EFO_0006506**	hepatic lipid content measurement	http://www.ebi.ac.uk/efo/EFO_0006506
**EFO_1001345**	Hepatitis, Alcoholic	http://www.ebi.ac.uk/efo/EFO_1001345
**EFO_0004612**	high density lipoprotein cholesterol measurement	http://www.ebi.ac.uk/efo/EFO_0004612
**EFO_0020866**	high density lipoprotein particle size change measurement	http://www.ebi.ac.uk/efo/EFO_0020866
**EFO_0008592**	high density lipoprotein particle size measurement	http://www.ebi.ac.uk/efo/EFO_0008592
**EFO_0004469**	HOMA-B	http://www.ebi.ac.uk/efo/EFO_0004469
**EFO_0004501**	HOMA-IR	http://www.ebi.ac.uk/efo/EFO_0004501
**HP_0003124**	hypercholesterolemia	http://purl.obolibrary.org/obo/HP_0003124
**MONDO_0021187**	hyperlipidemia	http://purl.obolibrary.org/obo/MONDO_0021187
**EFO_0000537**	hypertension	http://www.ebi.ac.uk/efo/EFO_0000537
**EFO_0004211**	hypertriglyceridemia	http://www.ebi.ac.uk/efo/EFO_0004211
**HP_0003146**	hypocholesterolemia	http://purl.obolibrary.org/obo/HP_0003146
**EFO_0005431**	illegal drug consumption	http://www.ebi.ac.uk/efo/EFO_0005431
**EFO_0004628**	insulin like growth factor measurement	http://www.ebi.ac.uk/efo/EFO_0004628
**EFO_0004467**	insulin measurement	http://www.ebi.ac.uk/efo/EFO_0004467
**EFO_0006830**	insulin metabolic clearance rate measurement	http://www.ebi.ac.uk/efo/EFO_0006830
**EFO_0008160**	insulin receptor measurement	http://www.ebi.ac.uk/efo/EFO_0008160
**EFO_0002614**	insulin resistance	http://www.ebi.ac.uk/efo/EFO_0002614
**EFO_0008473**	insulin response measurement	http://www.ebi.ac.uk/efo/EFO_0008473
**EFO_0008001**	insulin secretion measurement	http://www.ebi.ac.uk/efo/EFO_0008001
**EFO_0004471**	insulin sensitivity measurement	http://www.ebi.ac.uk/efo/EFO_0004471
**EFO_0020472**	insulin-degrading enzyme measurement	http://www.ebi.ac.uk/efo/EFO_0020472
**EFO_0020473**	insulin-like growth factor 1 receptor measurement	http://www.ebi.ac.uk/efo/EFO_0020473
**EFO_0020474**	insulin-like growth factor-binding protein 1 measurement	http://www.ebi.ac.uk/efo/EFO_0020474
**EFO_0020475**	insulin-like growth factor-binding protein 2 measurement	http://www.ebi.ac.uk/efo/EFO_0020475
**EFO_0020476**	insulin-like growth factor-binding protein 4 measurement	http://www.ebi.ac.uk/efo/EFO_0020476

**EFO_0020477**	insulin-like growth factor-binding protein 5 measurement	http://www.ebi.ac.uk/efo/EFO_0020477
**EFO_0020478**	insulin-like growth factor-binding protein 6 measurement	http://www.ebi.ac.uk/efo/EFO_0020478
**EFO_0008161**	insulin-like growth factor-binding protein 7 measurement	http://www.ebi.ac.uk/efo/EFO_0008161
**EFO_0009961**	Insulinogenic index measurement	http://www.ebi.ac.uk/efo/EFO_0009961
**EFO_0020906**	intermediate density lipoprotein change measurement	http://www.ebi.ac.uk/efo/EFO_0020906
**EFO_0008595**	intermediate density lipoprotein measurement	http://www.ebi.ac.uk/efo/EFO_0008595
**EFO_0009371**	ketogenic diet	http://www.ebi.ac.uk/efo/EFO_0009371
**EFO_0007804**	LDL cholesterol change measurement	http://www.ebi.ac.uk/efo/EFO_0007804
**EFO_0007930**	LDL cholesterol:HDL cholesterol ratio	http://www.ebi.ac.uk/efo/EFO_0007930
**EFO_0004995**	lean body mass	http://www.ebi.ac.uk/efo/EFO_0004995
**EFO_0009890**	lean mass-adjusted fat body mass	http://www.ebi.ac.uk/efo/EFO_0009890
**EFO_0010724**	lifestyle measurement	http://www.ebi.ac.uk/efo/EFO_0010724
**EFO_0020859**	lipid change measurement	http://www.ebi.ac.uk/efo/EFO_0020859
**EFO_0004529**	lipid measurement	http://www.ebi.ac.uk/efo/EFO_0004529
**EFO_0005105**	lipid or lipoprotein measurement	http://www.ebi.ac.uk/efo/EFO_0005105
**EFO_0007645**	longitudinal alcohol consumption measurement	http://www.ebi.ac.uk/efo/EFO_0007645
**EFO_0005937**	longitudinal BMI measurement	http://www.ebi.ac.uk/efo/EFO_0005937
**EFO_0004611**	low density lipoprotein cholesterol measurement	http://www.ebi.ac.uk/efo/EFO_0004611
**EFO_0020908**	low density lipoprotein particle size change measurement	http://www.ebi.ac.uk/efo/EFO_0020908
**EFO_0008593**	low density lipoprotein particle size measurement	http://www.ebi.ac.uk/efo/EFO_0008593
**EFO_0009946**	low density lipoprotein triglyceride measurement	http://www.ebi.ac.uk/efo/EFO_0009946
**EFO_0020538**	low-density lipoprotein receptor-related protein 8 measurement	http://www.ebi.ac.uk/efo/EFO_0020538
**EFO_0600067**	mastiha supplement exposure measurement	http://www.ebi.ac.uk/efo/EFO_0600067
**EFO_0005115**	metabolic rate measurement	http://www.ebi.ac.uk/efo/EFO_0005115
**EFO_0000195**	metabolic syndrome	http://www.ebi.ac.uk/efo/EFO_0000195
**EFO_0009382**	metabolically healthy obesity	http://www.ebi.ac.uk/efo/EFO_0009382
**EFO_0003095**	non-alcoholic fatty liver disease	http://www.ebi.ac.uk/efo/EFO_0003095
**EFO_0008421**	non-alcoholic fatty liver disease severity measurement	http://www.ebi.ac.uk/efo/EFO_0008421
**EFO_1001249**	non-alcoholic steatohepatitis	http://www.ebi.ac.uk/efo/EFO_1001249

**EFO_0010095**	non-grapefruit juice consumption measurement	http://www.ebi.ac.uk/efo/EFO_0010095
**EFO_0007931**	non-HDL cholesterol:HDL cholesterol ratio	http://www.ebi.ac.uk/efo/EFO_0007931
**EFO_0005689**	non-high density lipoprotein cholesterol measurement	http://www.ebi.ac.uk/efo/EFO_0005689
**EFO_0600066**	nutritional supplement exposure measurement	http://www.ebi.ac.uk/efo/EFO_0600066
**EFO_0007041**	obese body mass index status	http://www.ebi.ac.uk/efo/EFO_0007041
**EFO_0001073**	obesity	http://www.ebi.ac.uk/efo/EFO_0001073
**EFO_0003918**	obstructive sleep apnea	http://www.ebi.ac.uk/efo/EFO_0003918
**EFO_0020878**	omega-3 polyunsaturated fatty acid change measurement	http://www.ebi.ac.uk/efo/EFO_0020878
**EFO_0010119**	omega-3 polyunsaturated fatty acid measurement	http://www.ebi.ac.uk/efo/EFO_0010119
**EFO_0020879**	omega-6 polyunsaturated fatty acid change measurement	http://www.ebi.ac.uk/efo/EFO_0020879
**EFO_0005680**	omega-6 polyunsaturated fatty acid measurement	http://www.ebi.ac.uk/efo/EFO_0005680
**EFO_0010732**	omega-6:omega-3 polyunsaturated fatty acid ratio	http://www.ebi.ac.uk/efo/EFO_0010732
**EFO_0005935**	overweight body mass index status	http://www.ebi.ac.uk/efo/EFO_0005935
**EFO_0020620**	oxidized low-density lipoprotein receptor 1 measurement	http://www.ebi.ac.uk/efo/EFO_0020620
**EFO_0008000**	peak insulin response measurement	http://www.ebi.ac.uk/efo/EFO_0008000
**EFO_0007890**	pericardial adipose tissue measurement	http://www.ebi.ac.uk/efo/EFO_0007890
**EFO_0020903**	phospholipid change measurement	http://www.ebi.ac.uk/efo/EFO_0020903
**EFO_0004639**	phospholipid measurement	http://www.ebi.ac.uk/efo/EFO_0004639
**EFO_0003940**	physical activity	http://www.ebi.ac.uk/efo/EFO_0003940
**EFO_0008002**	physical activity measurement	http://www.ebi.ac.uk/efo/EFO_0008002
**EFO_0020880**	polyunsaturated fatty acid change measurement	http://www.ebi.ac.uk/efo/EFO_0020880
**EFO_0010733**	polyunsaturated fatty acid measurement	http://www.ebi.ac.uk/efo/EFO_0010733
**EFO_0000666**	portal hypertension	http://www.ebi.ac.uk/efo/EFO_0000666
**EFO_0007770**	post-operative fentanyl consumption measurement	http://www.ebi.ac.uk/efo/EFO_0007770
**EFO_0007632**	postprandial hyperlipidemia	http://www.ebi.ac.uk/efo/EFO_0007632
**MONDO_0001999**	primary pulmonary hypertension	http://purl.obolibrary.org/obo/MONDO_0001999
**EFO_0010810**	protein intake measurement	http://www.ebi.ac.uk/efo/EFO_0010810
**EFO_0001361**	pulmonary arterial hypertension	http://www.ebi.ac.uk/efo/EFO_0001361
**Orphanet_101953**	rare dyslipidemia	http://www.orpha.net/ORDO/Orphanet_101953

**EFO_0020867**	remnant cholesterol change measurement	http://www.ebi.ac.uk/EFO/EFO_0020867
**EFO_0004864**	renal sinus adipose tissue measurement	http://www.ebi.ac.uk/efo/EFO_0004864
**EFO_0005526**	response to alcohol	http://www.ebi.ac.uk/efo/EFO_0005526
**EFO_0005844**	response to dietary antigen	http://www.ebi.ac.uk/efo/EFO_0005844
**EFO_0005403**	response to dietary potassium supplementation	http://www.ebi.ac.uk/efo/EFO_0005403
**EFO_0600021**	response to dietary selenium supplementation	http://www.ebi.ac.uk/efo/EFO_0600021
**EFO_0007768**	response to exercise	http://www.ebi.ac.uk/efo/EFO_0007768
**EFO_0007684**	response to high fat food intake	http://www.ebi.ac.uk/efo/EFO_0007684
**EFO_0005401**	response to high sodium diet	http://www.ebi.ac.uk/efo/EFO_0005401
**EFO_0009372**	response to ketogenic diet	http://www.ebi.ac.uk/efo/EFO_0009372
**EFO_0009308**	response to long-chain n-3 PUFA dietary supplementation	http://www.ebi.ac.uk/efo/EFO_0009308
**EFO_0010731**	response to low calorie diet	http://www.ebi.ac.uk/efo/EFO_0010731
**EFO_0005402**	response to low sodium diet	http://www.ebi.ac.uk/efo/EFO_0005402
**EFO_0009131**	response to polyunsaturated fatty acid supplementation	http://www.ebi.ac.uk/efo/EFO_0009131
**EFO_0008396**	response to reward	http://www.ebi.ac.uk/efo/EFO_0008396
**GO_0006950**	response to stress	http://purl.obolibrary.org/obo/GO_0006950
**EFO_0009796**	response to supplemental oxygen	http://www.ebi.ac.uk/efo/EFO_0009796
**GO_0033273**	response to vitamin	http://purl.obolibrary.org/obo/GO_0033273
**EFO_0010648**	response to vitamin B3	http://www.ebi.ac.uk/efo/EFO_0010648
**EFO_0008004**	resting metabolic rate measurement	http://www.ebi.ac.uk/efo/EFO_0008004
**EFO_1002034**	secondary hypertension	http://www.ebi.ac.uk/efo/EFO_1002034
**EFO_0010818**	sensory perception of dietary content	http://www.ebi.ac.uk/efo/EFO_0010818
**EFO_0008343**	sex interaction measurement	http://www.ebi.ac.uk/efo/EFO_0008343
**EFO_0004807**	short sleep	http://www.ebi.ac.uk/efo/EFO_0004807
**EFO_0009825**	sign or symptom concerning food and fluid intake	http://www.ebi.ac.uk/efo/EFO_0009825
**EFO_0003877**	sleep apnea	http://www.ebi.ac.uk/efo/EFO_0003877
**EFO_0007817**	sleep apnea measurement	http://www.ebi.ac.uk/efo/EFO_0007817
**EFO_0008456**	sleep apnea measurement during non-REM sleep	http://www.ebi.ac.uk/efo/EFO_0008456
**EFO_0008455**	sleep apnea measurement during REM sleep	http://www.ebi.ac.uk/efo/EFO_0008455

**EFO_0005273**	sleep depth	http://www.ebi.ac.uk/efo/EFO_0005273
**EFO_0008568**	sleep disorder	http://www.ebi.ac.uk/efo/EFO_0008568
**EFO_0005271**	sleep duration	http://www.ebi.ac.uk/efo/EFO_0005271
**EFO_0005280**	sleep latency	http://www.ebi.ac.uk/efo/EFO_0005280
**EFO_0004870**	sleep measurement	http://www.ebi.ac.uk/efo/EFO_0004870
**EFO_0005272**	sleep quality	http://www.ebi.ac.uk/efo/EFO_0005272
**EFO_0005274**	sleep time	http://www.ebi.ac.uk/efo/EFO_0005274
**NCIT_C95746**	sleepiness	http://purl.obolibrary.org/obo/NCIT_C95746
**EFO_0004318**	smoking behavior	http://www.ebi.ac.uk/efo/EFO_0004318
**EFO_0005671**	smoking behaviour measurement	http://www.ebi.ac.uk/efo/EFO_0005671
**EFO_0004319**	smoking cessation	http://www.ebi.ac.uk/efo/EFO_0004319
**EFO_0005670**	smoking initiation	http://www.ebi.ac.uk/efo/EFO_0005670
**EFO_0006527**	smoking status measurement	http://www.ebi.ac.uk/efo/EFO_0006527
**EFO_0004622**	sphingolipid measurement	http://www.ebi.ac.uk/efo/EFO_0004622
**EFO_0004766**	subcutaneous adipose tissue measurement	http://www.ebi.ac.uk/efo/EFO_0004766
**EFO_0010158**	sugar consumption measurement	http://www.ebi.ac.uk/efo/EFO_0010158
**EFO_0010097**	sugar sweetened beverage consumption measurement	http://www.ebi.ac.uk/efo/EFO_0010097
**EFO_0010090**	sweet beverage consumption measurement	http://www.ebi.ac.uk/efo/EFO_0010090
**EFO_0006335**	systolic blood pressure	http://www.ebi.ac.uk/efo/EFO_0006335
**EFO_0006944**	systolic blood pressure change measurement	http://www.ebi.ac.uk/efo/EFO_0006944
**EFO_0010091**	tea consumption measurement	http://www.ebi.ac.uk/efo/EFO_0010091
**EFO_0007806**	total cholesterol change measurement	http://www.ebi.ac.uk/efo/EFO_0007806
**EFO_0004574**	total cholesterol measurement	http://www.ebi.ac.uk/efo/EFO_0004574
**EFO_0007678**	total fat intake measurement	http://www.ebi.ac.uk/efo/EFO_0007678
**EFO_0006823**	total trans-18:1 fatty acid measurement	http://www.ebi.ac.uk/efo/EFO_0006823
**EFO_0006821**	trans fatty acid measurement	http://www.ebi.ac.uk/efo/EFO_0006821
**EFO_0006825**	trans/cis-18:2 fatty acid measurement	http://www.ebi.ac.uk/efo/EFO_0006825
**EFO_0006826**	trans/trans-18:2 fatty acid measurement	http://www.ebi.ac.uk/efo/EFO_0006826
**EFO_0006822**	trans-16:1n-7 fatty acid measurement	http://www.ebi.ac.uk/efo/EFO_0006822

**EFO_1002006**	treatment-resistant hypertension	http://www.ebi.ac.uk/efo/EFO_1002006
**EFO_0007681**	triglyceride change measurement	http://www.ebi.ac.uk/efo/EFO_0007681
**EFO_0004530**	triglyceride measurement	http://www.ebi.ac.uk/efo/EFO_0004530
**EFO_0007929**	triglyceride:HDL cholesterol ratio	http://www.ebi.ac.uk/efo/EFO_0007929
**MONDO_0005147**	type 1 diabetes mellitus	http://purl.obolibrary.org/obo/MONDO_0005147
**MONDO_0005148**	type 2 diabetes mellitus	http://purl.obolibrary.org/obo/MONDO_0005148
**EFO_0005936**	underweight body mass index status	http://www.ebi.ac.uk/efo/EFO_0005936
**EFO_0010544**	uridine diphosphate glucose measurement	http://www.ebi.ac.uk/efo/EFO_0010544
**EFO_0006796**	very long-chain saturated fatty acid measurement	http://www.ebi.ac.uk/efo/EFO_0006796
**EFO_0020857**	very low density lipoprotein cholesterol change measurement	http://www.ebi.ac.uk/efo/EFO_0020857
**EFO_0008317**	very low density lipoprotein cholesterol measurement	http://www.ebi.ac.uk/efo/EFO_0008317
**EFO_0020907**	very low density lipoprotein particle size change measurement	http://www.ebi.ac.uk/efo/EFO_0020907
**EFO_0008594**	very low density lipoprotein particle size measurement	http://www.ebi.ac.uk/efo/EFO_0008594
**EFO_0004765**	visceral adipose tissue measurement	http://www.ebi.ac.uk/efo/EFO_0004765
**EFO_0004767**	visceral:subcutaneous adipose tissue ratio	http://www.ebi.ac.uk/efo/EFO_0004767
**EFO_0009701**	visceral:total adipose tissue ratio	http://www.ebi.ac.uk/efo/EFO_0009701
**EFO_0000734**	vitamin b12 deficiency	http://www.ebi.ac.uk/efo/EFO_0000734
**EFO_0003762**	vitamin D deficiency	http://www.ebi.ac.uk/efo/EFO_0003762
**EFO_0008539**	vitamin D dietary intake measurement	http://www.ebi.ac.uk/efo/EFO_0008539
**EFO_0009116**	vitamin supplement exposure measurement	http://www.ebi.ac.uk/efo/EFO_0009116
**EFO_0004342**	waist circumference	http://www.ebi.ac.uk/efo/EFO_0004342
**EFO_0005191**	waist height ratio	http://www.ebi.ac.uk/efo/EFO_0005191
**EFO_0004343**	waist-hip ratio	http://www.ebi.ac.uk/efo/EFO_0004343

Supplementary Table 2: Selected GWAS studies with interaction with obesity traits

Author (Journal, Year) [Ref]	Gene / near gene	SNP	Phenotype	Environmental factor	Covariates included in the model	Ancestry	Sample size	Comments
**Wang H (Sci Adv, 2019) [1]**	FTO	rs11642015	BMI	PA, SB & smoking	Raw phenotype values were adjusted for age and the first 10 PCs	European	347,158 individuals	-
	BCDIN3D	rs7132908	BMI	SB				
	FAM150B	rs62104180	WC	PA				
	FTO	rs1421085	WC	PA & SB				
**Jung HU (Sci Rep, 2021) [2]**	EFNB2	rs2391331	BMI	7environmental factors *	Age, sex, and recruitment area	Korean	8,155 individuals	* 7environmental factors including alcohol, education, income, total calorie intake, protein intake, carbohydrate intake and smoking
**Fujihara K (Nutrients, 2021) [3]**	TXNRD1	rs4445711	BMI	Carrot intake	Age, sex	Japanese	12,225 individuals	Interaction was attenuated after adjustment for age, sex, alcohol intake, smoking, physical activity and the frequency of total vegetable intake (p = 2.1 × 10−7)
**Smith CE (Mol Nutr Food Res, 2018) [4]**	LINC00333	rs9635058	BMI	Dairy intake	Model 1: Age, sex, field center and familial relationships and principal components. Model 2: Model 1 covariates + total	European	Up to 25,513 individuals	-

					energy, physical activity and the CHARGE diet score			
**Graff M (PLoS Genet, 2017) [5]**	CDH12	rs986732	BMI	PA	Age, age2, sex and cohort	African American, European, Filipino, and Indian	156,653 individuals	-
	FTO	rs9941349	BMI	PA				
	MRAS	rs1720825	BMI	PA				
	ELAVL2	rs1934100	BMI	PA				
	CCK	rs754635	BMI	PA				
	ZSCAN2	rs7176527	WCadjBMI	PA				
	PAPPA2	rs4650943	WHRadjBMI	PA				
	MEIS1	rs2300481	WHRadjBMI	PA				
	ARHGEF28	rs167025	WHRadjBMI	PA				
	HCP5	rs3094013	WHRadjBMI	PA				
	BAZ1B	rs6976930	WHRadjBMI	PA				
	PLCE1	rs10786152	WHRadjBMI	PA				
	CTRB2	rs889512	WHRadjBMI	PA				
**Park S (Nutrients, 2021) [6]**	PRS	*	BMI	Plant based diet	Age, gender, education, income, energy intake, occupation, residence area, regular exercise, alcohol intake, and smoking status	Korean	17,545 cases & 36,283 controls	* PRS including rs543874 (SEC16B), rs713586 (DNAJC27), rs6265 (BDNF), rs6567160 (MC4R) and rs1444988703 (GIPR)
			BMI	Fried foods				

**Ahmad S (Int J Obes (Lond), 2016) [7]**	FLJ33534	rs140133294	BMI	Smoking	Age, age2, sex and the first 10 principal components to account for population stratification.	Pakistani	14,131 individuals	-
**Justice AE (Nat Commun, 2017) [8]**	INPP4B	rs336396	BMI	Smoking	Sex, age and age2, and (when appropriate) for study site and principal components to account for ancestry	African American/Afro-Caribbean, European, Indian Asian, Filipino, and Hispanic/Latino	196,760 individuals	-
	CHRNB4	rs12902602	BMI	Smoking				
	ADAMTS7	rs3813565	BMI	Smoking				
	GRIN2A	rs4141488	WCadjBMI	Smoking				
	LYPLAL1	rs765751	WHRadjBMI	Smoking				
	RSPO3	rs7766106	WHRadjBMI	Smoking				

PA: Physical activity; SB: Sweetened Beverages; BMI: Body Mass Index; WC: Waist Circumference; WCadjBMI: Waist Circumference adjusted for BMI; WHRadjBMI: Waist-hip ratio adjusted for BMI

Supplementary Table 3: Selected GWAS studies with interaction with glucose metabolism traits

Author (Journal, Year) [Ref]	Gene / near gene	SNP	Phenotype	Environmental factor	Covariates included in the model	Ancestry	Sample size	Comments
**Daily JW (Nutr Metab Cardiovasc Dis, 2018) [9]**	GRS	*	HOMA-B	Coffee consumption	adjusting for covariates such as age, gender, residence area, and BMI with and without alcohol consumption.	Korean	8,842 individuals	* GRS including: rs2974430 (SLIT3), rs1077044 (PLEKHA5), and rs16838853 (PPP2R2C)
**Jin T (Nutrients, 2020) [10]**	wGRS	*	Incidence of prediabetes	Coffee consumption	Adjusted for age, sex, BMI, smoking status, alcohol consumption, family history of type 2 diabetes and total energy intake (kcal/day, continuous)	Korean	6,080 cases & 1,788 controls	* wGRS including rs2074356 (HECTD4), rs11066015 (ACAD10), rs12229654 (MYL2), rs11065828 (CUX2), and rs79105258 (CUX2) (in 12q24.11-13)
**Zheng JS (PLoS One, 2013) [11]**	ISCA1L||HTR1A	rs16891077	Insulin	Carbohydrate	Adjusted for age, sex, study center, kinship and population structure and family relationship as a random effect.	European	820 individuals	**Only SNP with p-value <5*10^-8 are showed in this table out of the total of 139 genes reported with nominal p-value <5*10^-5
	ISCA1L||HTR1A	rs16891077	HOMA-IR	Carbohydrate				
	ISCA1L||HTR1A	rs4700512	Insulin	Carbohydrate				
	ISCA1L||HTR1A	rs4700512	HOMA-IR	Carbohydrate				
	LOC100132037	rs2421332	HOMA-B	n-6 PUFA				
	ISCA1L||HTR1A	rs4700021	Insulin	Carbohydrate				
	ISCA1L||HTR1A	rs4700021	HOMA-IR	Carbohydrate				
	EMX2	rs7088969	HOMA-B	n-6 PUFA				
	NRG3	rs9787485	HOMA-IR	Carbohydrate				
	NRG3	rs9787485	Insulin	Carbohydrate				
	ISCA1L	rs7709357	Insulin	Carbohydrate				

	ISCA1L	rs10078232	Insulin	Carbohydrate				
	ISCA1L	rs10059856	Insulin	Carbohydrate				
	ISCA1L	rs10058718	Insulin	Carbohydrate				
**Kim J (PLoS One, 2017) [12]**	JMJD1C	rs10761745	T2D cases	Iron intake (in men)	Step1 (screening): adjusting for age, sex, BMI, and residential area. Step 2: Aged adjusted linear model with trend across iron intake or hemoglobin levels groups	Korean	911 prevalent diabetes cases & 7,024 incident diabetes and non-diabetes controls	-
	KCNQ1	rs163177	T2D cases	Iron intake (in women)				
**Franck M (Lifestyle Genom, 2020) [13]**	GRS	*	HOMA-IR	n-3 PUFA supplementation	NA	French Canadian	32 cases & 106 controls	GRS including rs72723587 (ADGRL2), rs77850702 (LOC101929563), rs72703546 (LOC101929563), rs17174795 (PTPRO), rs12437986 (TPM1), rs35621498 (RPH3AL), rs55842940 (RPH3AL), rs6001872 (TNRC6B)

GRS: Genetic Risk Score; wGRS: weighted Genetic Risk Score

Supplementary Table 4: Selected GWAS studies with interaction with blood pressure traits

Author (Journal, Year) [Ref]	Gene / near gene	SNP	Phenotype	Environmental factor	Covariates included in the model	Ancestry	Sample size	Comments
**Simino J (Front Genet, 2013) [14]**	SLC16A9	rs10826334	SBP	Alcohol consumption	Step 1: age, sex, BMI, and antihypertensive medication. For the joint 2 df test they did not perform any adjustment on the test statistics.	European	Up to 6,882 individuals	-
**Feitosa MF (PLoS One, 2018) [15]**	SGK223	rs10092965	DBP	Current Drinkers	Age, sex, principal components (PCs), and other study-specific covariates	European, African American or Afro-Caribbean, Asian, and Hispanic or Latin American	81,327 drinkers & 52,026 non-drinkers	Genes associated with BP traits in Multi-ancestry meta-analysis in combined Stage 1 and Stage 2.
	SGK223	rs7823056	PP	Light vs Heavy Drinkers				
	SGK223	rs7823056	SBP	Light vs Heavy Drinkers				
	PPP1R3B	rs453301	DBP	Current Drinkers				
	LOC157273	rs10503387	SBP	Current Drinkers				
	LOC157273	rs11781008	DBP	Current Drinkers				
	TNKS	rs4383974	SBP	Current Drinkers				
	TNKS	rs9286060	DBP	Current Drinkers				
	MSRA	rs34919878	DBP	Current Drinkers				
	MSRA	rs4841294	SBP	Light vs Heavy Drinkers				
	MSRA	rs17693945	MAP	Light vs Heavy Drinkers				
	PINX1	rs13276026	DBP	Current Drinkers				
	PINX1	rs13276026	MAP	Current Drinkers				
	PINX1	rs13276026	SBP	Current Drinkers				
	PINX1	rs4551304	DBP	Current Drinkers				
	PINX1	rs4551304	MAP	Current Drinkers				
	XKR6	rs9969436	MAP	Light vs Heavy Drinkers				

	BLK	rs2409784	DBP	Current Drinkers				
	LINC00208	rs2244894	PP	Current Drinkers				
	LINC00208	rs13249843	DBP	Current Drinkers				
	GATA4	rs3735814	SBP	Current Drinkers				
	FTO	rs9928094	PP	Current Drinkers				
	FTO	rs62033406	MAP	Current Drinkers				
**Kim Y (Genet Epidemiol, 2020) [16]**	LGR5	rs1297184	Blood pressure	Alcohol consumption	Age, sex, area, smoking status, physical activity, marital status, Na/K consumption ratio, and WC.	Korean	6,176 individuals	-
**Lin WY (Front Genet, 2018) [17]**	ADAMTS7P1	rs74249839	DBP	Alcohol consumption	Age, gender, BMI, and the first seven PCs	Han Chinese and unknown ancestry	1,764 alcohol drinkers & 14,779 non-drinkers	-
	ADAMTS7P1	rs16973457	DBP & SBP	Alcohol consumption				
	ADAMTS7P1	rs4238534	DBP & SBP	Alcohol consumption				
**Lin WY (Brief Bioinform, 2019) [18]**	MIR31HG	rs10811568	DBP	Alcohol consumption	adjusting for age, gender, BMI, and the first seven principal components.	East Asian	1,764 alcohol-drinking individuals & 14,779 non-alcohol-drinking individuals	-
	CEP112	rs62065089	SBP	Alcohol consumption				
	ACYP2	rs79990035	DBP	Smoking habit				
**He J (Circ Cardiovasc Genet, 2013) [19]**	PRMT6	rs1330225	Blood Pressure	During Low-Sodium Intervention	family structure and adjust for age, gender, and body-mass index (BMI)	Han Chinese	1,881 individuals	* Baseline
	CDCA7	rs10930597	Blood Pressure	During Low-Sodium Intervention				
	PIBF1	rs8002688	Blood Pressure	Responses to Low-Sodium				

	IRAK1BP1	rs16890334	Blood Pressure	During High-Sodium Intervention				
	CDCA7	rs10930597	Blood Pressure	During Potassium Intervention				
	ARL4C	rs11887188	Blood Pressure	During Potassium Intervention				
	IRAK1BP1	rs16890334	Blood Pressure	During Potassium Intervention				
	SALL1	rs2030114	Blood Pressure	During Potassium Intervention				
	TRPM8	rs7577262	Blood Pressure	Response to Cold Pressor Test *				
	FBXL13	rs17135875	Blood Pressure	Response to Cold Pressor Test *				
**Osazuwa-Peters OL (Genet Epidemiol, 2020) [20]**	Intergenic	rs969219	DBP	Dichotomous lifestyle risk score	the vector of covariates including age, sex, lifestyle exposure interactions with age (age*E) and sex (sex*E).	European	1,924 unexposed individuals & 4,330 exposed individuals	**Only SNP with p-value <5*10^-8 are showed in this table out of the total of 22 genes reported with nominal p-value <5*10^-6 Lifestyle risk score summarizing Smoking + Alcohol intake + Education + Physical Activity
	Intergenic	rs11131920	DBP	Dichotomous lifestyle risk score				
	Intergenic	rs11131921	DBP	Dichotomous lifestyle risk score				
**Sung YJ (Am J Hypertens, 2015)**	LRP2	rs2268365	SBP	Pack-years	Age, sex, body mass index, and antihypertensive	European	Up to 6,889 individuals	**Only SNP with p-value <5*10^-8 are showed in this table out of the
	MECOM	rs12634933	SBP	Pack-years				

[21]	COLEC10-MAL2	rs6989684	SBP	Pack-years	medication use (yes/no) were included as covariables			total of 28 genes reported with nominal p-value <5*10^-6 Pack-years are calculated as the average number of packs smoked per day times the total number of years a subject smoked during his/her lifetime.
	TRAPPC9	rs7823724	SBP	Pack-years				
	CYB5B	rs12149862	SBP	Pack-years				
	Intergenic	rs4573996	SBP	Cigarettes per day				
	MN1	rs133980	SBP	Cigarettes per day				
**Sung YJ (Am J Hum Genet, 2018) [22]**	NPPA;NPPB	rs12741980	SBP	Smoking exposure	age, sex, field center (for multi-center studies), and principal component (PC) (to account for population stratification and admixture)	European, African, Asian, and Hispanic	23,685 current smokers & 106,216 former and never smokers	Genes associated with BP traits in combined Trans-ancestry meta-analysis of Stage 1 and Stage 2.
	RSRC1	rs201851995	SBP	Smoking exposure				
	INPP4B;GAB1	rs78763922	SBP	Smoking exposure				
	HOTTIP	rs2023843	SBP	Smoking exposure				
	MFHAS1	rs201133964	SBP	Smoking exposure				
	PPP1R3B;TNKS	rs35904419	DBP	Smoking exposure				
	FAM167A-AS1	rs4841531	SBP	Smoking exposure				
	EBF2	rs58429174	DBP	Smoking exposure				
	ADRB1	rs180940	DBP	Smoking exposure				
	AP5B1;OVOL1	rs201316070	SBP	Smoking exposure				
	LRP6	rs72656645	SBP	Smoking exposure				
	SLCO1C1	rs73073686	DBP	Smoking exposure				
	ATP2B1	rs10858948	DBP	Smoking exposure				
	MED13L	rs11067762	DBP	Smoking exposure				
	CYP1A1-2	rs10628234	DBP	Smoking exposure				
	LDHD	rs4888411	SBP	Smoking exposure				
	SLC2A4	rs9899183	SBP	Smoking exposure				
	ACE	rs4968782	DBP	Smoking exposure				

**Taylor JY (Sci Rep, 2016) [23]**	NEDD8	rs11158609	SBP	Cigarette smoking	Age, age2, BMI and top 10 principal components (PCs) of the GWAS data	African American	1,083 individuals	-
	TTYH2	rs8078051	SBP	Cigarette smoking				
**Hachiya T (Sci Rep, 2018) [24]**	BCL11B	rs8022678	SBP	Daily sodium consumption	Population stratification (20 principal components), age, sex, and BMI	Japanese	4,527 individuals	-
**Li C (Circ Cardiovasc Genet, 2017) [25]**	ARL15	rs16882447	SBP	Dietary potassium intake	Age, gender, and BMI. Principal components analysis revealed population substructure in MESA (but not GenSalt). Therefore, ancestry was also accounted for in MESA by adjusting for the first three principal components.	Han Chinese	1,876 individuals	++Discovery stage gene-base analysis
	RANBP3L	rs958929	PP	Dietary potassium intake				
	CC2D2A	++	SBP	Dietary potassium intake				
	BNC2	++	SBP	Dietary potassium intake				
	GGNBP1	++	DBP	Dietary potassium intake				
	LINC00336	++	DBP	Dietary potassium intake				
	DAB1	++	PP	Dietary potassium intake				
	MIR4466	++	PP	Dietary potassium intake				
	BNC2	++	MAP	Dietary potassium intake				
**Li C (Hypertension, 2016) [26]**	UST	rs13211840	DBP	Dietary sodium intake	Age, gender, and BMI. Principal components analysis revealed	Han Chinese	1,876 individuals	-
	CLGN	rs2567241	SBP	Dietary sodium intake				
	LOC105369882	rs11104632	SBP	Dietary sodium intake				
	CLGN	rs2567241	DBP	Dietary sodium intake				

	CLGN	rs2567241	MAP	Dietary sodium intake	population substructure in MESA (but not GenSalt). Therefore, ancestry was also accounted for in MESA by adjusting for the first three principal components.			
	MKNK1	++	SBP	Dietary sodium intake				
	C2orf80	++	PP	Dietary sodium intake				
	EPHA6	++	PP	Dietary sodium intake				
	SCOC-AS1	++	DBP & MAP	Dietary sodium intake				
	SCOC	++	SBP & DBP & MAP	Dietary sodium intake				
	CLGN	++	SBP & DBP & MAP	Dietary sodium intake				
	MGAT4D	++	SBP & MAP	Dietary sodium intake				
	ARHGAP42	++	DBP	Dietary sodium intake				
	CASP4	++	MAP	Dietary sodium intake				
	LINC01478	++	SBP	Dietary sodium intake				
**Park YM (Nutrients, 2017) [27]**	CSK	rs1378942	Hypertension	24 h urinary Na-K ratio	Cochran-Mantel-Haenzel analysis were applied to determine potential confounders	Korean	8,839 individuals	-
	CSK-MIR4513	rs3784789	Hypertension	24 h urinary Na-K ratio				
**Jeong H (Nutrients, 2020) [28]**	FGF5	rs16998073	Hypertension	Dietary sodium intake	Sex, age, examination site, smoking status, alcohol consumption, physical activity, and BMI to minimize the effect of confounding factors	Korean	8,178 cases & 9,558 controls	-
	FGF5	rs16998073	Hypertension	Dietary potassium intake				
	FGF5	rs16998073	Hypertension	Dietary NA-K ratio				

SBP: Systolic Blood Pressure; DBP: Diastolic Blood Pressure; BP: Blood Pressure; MAP: Mean Arterial Pressure; PP: Pulse Pressure

Supplementary Table 5: Selected GWAS studies with interaction with cholesterol traits

Author (Journal, Year) [Ref]	Gene / near gene	SNP	Phenotype	Environmental factor	Covariates included in the model	Ancestry	Sample size	Comments
**Francis M (PLoS Genet, 2021) [29]**	MLXIPL	rs799157	LDL	Fish oil supplementation	age, sex, body mass index (BMI), weekly servings of oily fish, socioeconomic status measured by Townsend deprivation index, and the first ten genetic principal components	European	73,962 individuals	-
	ABCA6	rs77542162	LDL	Fish oil supplementation				
	SLC12A3	rs148931404	HDL	Fish oil supplementation				
**An P (Hum Genet, 2014) [30]**	LOC100420502	rs927969	Posrprandial NHDL	High Fat meal	adjusted for age, age2, age3, BMI, baseline NHDL, centers and 10 PCs, by sex	European	928 individuals	-
	CDH13	rs17756260	Posrprandial NHDL	High Fat meal				
**Nishida Y (J Lipid Res, 2020) [31]**	ABCA1	rs1883025	HDL	Physical activity	adjustment for age, investigation site, BMI, alcohol drinking, and cigarette smoking.	Japanese	2,231 men	Only one interaction of ABCA1 rs1883025 with PA was statistically significant in men, after Bonferroni correction [P-interaction = 0.001 (α = 0.05/21 = 0.002)]

Kilpeläinen TO (Nat Commun, 2019) [32]	CLASP1	rs2862183	HDL	Physical activity	covariates which included age, sex, study center (for multi-center studies), and genome-wide principal components.	African American, Asian, European, and Hispanic or Latin American	34,541 inactive individuals & 75,147 active individuals	
	LHX1	rs295849	HDL	Physical activity				
	SNTA1	rs141588480	HDL	Physical activity				
	CNTNAP2	rs190748049	LDL	Physical activity				
Liu M (Clin Nutr, 2020) [33]	Haplotype	*	HDL	Protein intake	Model 1 included the covariates of residence area, gender, age, and BMI. The model 2 included adjustments for residence area, gender, age, BMI, smoking status, coffee intake, drinking status, total physical activity, and serum total cholesterol concentration	Korean	2,873 cases & 5,966 controls	* Haplotype including PTPN11_rs11066325, RPH3A_rs886477 and OAS3_rs2072134
	Haplotype	*	HDL	Fat intake				
	Haplotype	*	HDL	Saturated Fat intake				
	Haplotype	*	HDL	Polyunsaturated Fat intake				
Noordam R (Nat Commun, 2019) [34]	FAM47E	rs1348206	HDL	Long total sleep time	All models were adjusted for age, sex, field centre (if	African American, Chinese, Asian,	12,758 short sleepers &	**Only novel loci are showed in this table out of the total of 288 genes reported in the
	MIR4280	rs2544681	HDL	Long total sleep time				
	ATP6V0A4	rs7799249	HDL	Long total sleep time				
	ATP6V1H	rs7462612	HDL	Long total sleep time				

	MIR331	rs4296102	HDL	Long total sleep time	required), and the first principal components to correct for population stratification.	European, Hispanic/Latino	48,790 controls	article (related with cholesterol traits)
	ARNT2	rs12593988	HDL	Long total sleep time				
	KIAA0195	rs6501801	HDL	Long total sleep time				
	FOXD2	rs4926854	LDL	Long total sleep time				
	NR5A2	rs2821357	LDL	Long total sleep time				
	BOC	rs1466848	LDL	Long total sleep time				
	IGFBP7-AS1	rs4075349	LDL	Long total sleep time				
	S100A6	rs6672390	HDL	Short total sleep time				
	SMARCAL1	rs1111341	HDL	Short total sleep time				
	FHIT	rs2594136	HDL	Short total sleep time				
	EPHB1	rs12695617	HDL	Short total sleep time				
	SVILP1	rs903269	HDL	Short total sleep time				
	CLEC2D	rs2080208	HDL	Short total sleep time				
	RGMA	rs4778087	HDL	Short total sleep time				
	ZNF827	rs7679068	LDL	Short total sleep time				
	KLHL31	rs681554	LDL	Short total sleep time				
	MAGI2	rs10244093	LDL	Short total sleep time				
	FUT10	rs4733156	LDL	Short total sleep time				
	SNX29	rs12598569	LDL	Short total sleep time				
	VAT1L	rs429921	LDL	Short total sleep time				
	GPRC5C	rs9906564	LDL	Short total sleep time				
	METRNL	rs8077967	LDL	Short total sleep time				
**Bentley AR (Nat Genet, 2019) [35]**	LOC105378783	rs12740061	HDL	Current Smoking	All models were adjusted for age, sex, field center and principal components	African, Asian, European, and Hispanic	Up to 125,692 individuals	-
	PTPRZ1	rs77810251	HDL	Ever-Smoking				
	CNTNAP2	rs73453125	LDL	Current Smoking				

	PRKAG2	rs56167574	LDL	Ever-Smoking	derived using genotyped SNPs.			
	MIR4686	rs79950627	LDL	Current Smoking				
**de Vries PS (Am J Epidemiol, 2019) [36]**	MACROD1	rs190528931	HDL	Current Drinkers	age, sex, ancestry-informative principal components, and study-specific variables where appropriate (such as center for multicenter studies)	European, African American, Asian, Hispanic	73,864 current drinkers & 55,532 non-drinkers	Of the 147 identified genome-wide-significant loci, 18 are novel lipid loci that have not been previously identified by other association studies for HDL-C, LDL-C, TG, or total cholesterol SNPs from trans-ancestry meta-analysis unless: * Only for African ancestry; **Only for European ancestry
	ETV5	rs80080062	HDL	Regular drinkers				
	TRIM2	rs72729610	HDL	Regular drinkers				
	ASB3/GPR75-ASB3	rs2111622**	HDL	Current Drinkers				
	DNAJC22/SPATS2	rs4898521**	HDL	Regular drinkers				
	C10orf88	rs7904973	LDL	Current Drinkers				
	CREB3L2	rs73729083	LDL	Current Drinkers				
	AGPAT5	rs2911971	LDL	Current Drinkers				
	PCSK5	rs7035578**	LDL	Current Drinkers				
	SMC6	rs201445483	LDL	Current Drinkers				
	SPRY1/LINC01091	rs143528679*	LDL	Current Drinkers				
	ZER1	rs13284665**	LDL	Current Drinkers				
**Rudkowska I (J Nutrigenet Nutrigenomics, 2015) [37]**	ABCG2	rs2622604	LDL-PPD	Fat intake	adjusted for age (years), age squared, gender, energy intake (kcal), TG (mmol/l), and BMI	European	541 individuals	Only 14 (shown in table) out of the total of the 29 SNPs founds were related to known genes.
	CPA3	rs3819340	LDL-PPD	Fat intake				
	FNBP1	rs10760649	LDL-PPD	Fat intake				
	FNBP1	rs806861	LDL-PPD	Fat intake				
	FNBP1	rs4837446	LDL-PPD	Fat intake				
	KCNQ3	rs2597327	LDL-PPD	Fat intake				
	LOC728503	rs303795	LDL-PPD	Fat intake				
	NBAS	rs17668738	LDL-PPD	Fat intake				
	NCALD	rs501344	LDL-PPD	Fat intake				
	NKAIN2	rs587706	LDL-PPD	Fat intake				
	OPRL1	rs2229205	LDL-PPD	Fat intake				
	SH3BGRL2	rs2295015	LDL-PPD	Fat intake				

	SOX5	rs12579036	LDL-PPD	Fat intake
	SUSD4	rs9442041	LDL-PPD	Fat intake

NHDL: Non-HDL cholesterol; LDL-PPD: Low-density lipoprotein peak particle diameter

Supplementary Table 6: Selected GWAS studies with interaction with triglycerides traits

Author (Journal, Year) [Ref]	Gene / near gene	SNP	Phenotype	Environmental factor	Covariates included in the model	Ancestry	Sample size	Comments
**Francis M (PLoS Genet, 2021) [29]**	GJB6	rs112803755	Triglycerides	Fish oil supplementation	Age, sex, body mass index (BMI), weekly servings of oily fish, socioeconomic status measured by Townsend deprivation index, and the first ten genetic principal components	European	73,962 individuals	-
**de Vries PS (Am J Epidemiol, 2019) [36]**	GAS6-AS1	rs7140110	Triglycerides	Current Drinkers	Age, sex, ancestry-informative principal components, and study-specific variables where appropriate (such as center for multicenter studies)	European, African American, Asian, Hispanic	73,864 current drinkers & 55,532 non-drinkers	Of the 147 identified genome-wide-significant loci, 18 are novel lipid loci that have not been previously identified by other association studies for HDL-C, LDL-C, TG, or total cholesterol SNPs from trans-ancestry meta-analysis unless: * Only for African ancestry; **Only for European ancestry
	TMEM175	rs34311866*	Triglycerides	Current Drinkers				
	HSPA4/FSTL4	rs56076449	Triglycerides	Regular drinkers				
	A1CF	rs41274050*	Triglycerides	Regular drinkers				
	EYA2	rs6063050	Triglycerides	Current Drinkers				
	LOC101927697/EBF1	rs2963472*	Triglycerides	Regular drinkers				

**Tan A (Hum Mol Genet, 2011) [38]**	ALDH2	rs671	Triglycerides	Alcohol consumption	Age, BMI and cigarette smoking.	Han Chinese	1,999 male individuals	-
**Wojczynski MK (Metabolism, 2015) [39]**	-	rs10243693	Postprandial triglycerides	High fat meal	Model 1: Within each sex strata, we used a stepwise regression approach, offering age, age^2,age^3, and 10 principal components and Model 1 + baseline TG	European	872 individuals	The study also found other 109 SNP-phenotype associations (85 unique SNPs) were considered 'suggestive' (p<1E-05) across all phenotypes.
	ZPR1	rs964184	Postprandial triglycerides	High fat meal				
**Sarzynski MA (Br J Sports Med, 2015) [40]**	DOCK10	rs2396190	Triglycerides	Exercise training	Age age^2,age^3, baseline BMI, and baseline TG using a stepwise regression procedure	European	478 individuals from 99 families	
	CYYR1	rs222158	Triglycerides	Exercise training				
	DCC	rs3906453	Triglycerides	Exercise training				
	CRTC3	rs3862435	Triglycerides	Exercise training				
	CRTC3	rs3862436	Triglycerides	Exercise training				
	PRRX2	rs3861882	Triglycerides	Exercise training				
	GPATCH2	rs2646822	Triglycerides	Exercise training				
	GPATCH2	rs2646817	Triglycerides	Exercise training				
**Coltell O (Nutrients, 2020) [41]**	ME1	rs3798890	serum omega-3 PUFA concentrations	Adherence to MedDiet	Model1: Sex and age<br>Model2: sex, age, and diabetes	Mediterranean	248 individuals with low Med diet adherence & 178 individuals with high	

							Med diet adherence	
**Park S (J Acad Nutr Diet, 2020) [42]**	APOA5	rs662799	Triglycerides	Fat intake	Year, residence area, age, sex, body mass index, smoking status, drinking status, coffee intake, daily activity, and plasma total cholesterol (TC) and serum glucose concentrations except when the interaction parameter was the same as the adjustment parameter	Korean	21,014 individuals with TG < 150mg/dL & 7,424 individuals with TG ≥ 150 mg/dL	
	APOA5	rs662799	Triglycerides	Carbohydrate intake				
	APOA5	rs662799	Triglycerides	Calcium intake				
	APOA5	rs662799	Triglycerides	Alcohol intake				
	APOA5	rs662799	Triglycerides	Smoking status				
	APOA5	rs2266788	Triglycerides	Fat intake				
	APOA5	rs2266788	Triglycerides	Carbohydrate intake				
	APOA5	rs2266788	Triglycerides	Alcohol intake				
	APOA5	rs2266788	Triglycerides	Smoking status				
**Noordam R (Nat Commun, 2019) [34]**	RNU5F-1	rs1857237	Triglycerides	Long total sleep time	Age, sex, field centre (if required), and the first principal components to correct for population stratification.	African American, Chinese, Asian, European, Hispanic/Latino	12,758 short sleepers & 48,790 controls	**Only novel loci are showed in this table out of the total of 113 genes reported in the article (related with triglyceride traits)
	SLC35F3	rs2801439	Triglycerides	Long total sleep time				
	MIR4790	rs6800190	Triglycerides	Long total sleep time				
	OSBPL10	rs6550067	Triglycerides	Long total sleep time				
	PDE3A	rs7965852	Triglycerides	Long total sleep time				
	ADAMTS17	rs8041815	Triglycerides	Long total sleep time				
	SULT2A1	rs296363	Triglycerides	Long total sleep time				
	RP4-660H19.1	rs10789347	Triglycerides	Short total sleep time				
	AC092635.1	rs1606045	Triglycerides	Short total sleep time				

	YPEL5	rs1447523	Triglycerides	Short total sleep time			
	AC097499.1	rs4849021	Triglycerides	Short total sleep time			
	PCDH18	rs10019234	Triglycerides	Short total sleep time			
	LINC01340	rs291821	Triglycerides	Short total sleep time			
	MIR548M	rs2714658	Triglycerides	Short total sleep time			
	DEFB136	rs11774568	Triglycerides	Short total sleep time			
	LINC01289	rs970908	Triglycerides	Short total sleep time			
	METTL15	rs7924896	Triglycerides	Short total sleep time			
	TMEM132B	rs10744213	Triglycerides	Short total sleep time			
	ACSM2B	rs11648341	Triglycerides	Short total sleep time			
	MYO9B	rs3826692	Triglycerides	Short total sleep time			
	TMX4	rs1058029	Triglycerides	Short total sleep time			
	MICAL3	rs5746495	Triglycerides	Short total sleep time			
**Bentley AR (Nat Genet, 2019) [35]**	ZNF729	rs60029395	Triglycerides	Current Smoking	Age, sex, field center and principal components derived using genotyped SNPs.	African, Asian, European, and Hispanic	Up to 125,692 individuals
	DGCR8	rs7364132	Triglycerides	Ever-Smoking			

Bibliograph

1. Wang H, Zhang F, Zeng J, Wu Y, Kemper KE, Xue A, et al. Genotype-by-environment interactions inferred from genetic effects on phenotypic variability in the UK Biobank. Sci Adv. 2019;5(8):eaaw3538. doi: 10.1126/sciadv.aaw3538.
2. Jung HU, Lee WJ, Ha TW, Kang JO, Kim J, Kim MK, et al. Identification of genetic loci affecting body mass index through interaction with multiple environmental factors using structured linear mixed model. Sci Rep. 2021;11(1):5001. doi: 10.1038/s41598-021-83684-1.
3. Fujihara K, Nogawa S, Saito K, Horikawa C, Takeda Y, Cho K, et al. Carrot Consumption Frequency Associated with Reduced BMI and Obesity through the SNP Intermediary rs4445711. Nutrients. 2021;13(10). doi: 10.3390/nu13103478.
4. Smith CE, Follis JL, Dashti HS, Tanaka T, Graff M, Fretts AM, et al. Genome-Wide Interactions with Dairy Intake for Body Mass Index in Adults of European Descent. Mol Nutr Food Res. 2018;62(3). doi: 10.1002/mnfr.201700347.
5. Graff M, Scott RA, Justice AE, Young KL, Feitosa MF, Barata L, et al. Genome-wide physical activity interactions in adiposity - A meta-analysis of 200,452 adults. PLoS Genet. 2017;13(4):e1006528. doi: 10.1371/journal.pgen.1006528.
6. Park S, Yang HJ, Kim MJ, Hur HJ, Kim SH, Kim MS. Interactions between Polygenic Risk Scores, Dietary Pattern, and Menarche Age with the Obesity Risk in a Large Hospital-Based Cohort. Nutrients. 2021;13(11). doi: 10.3390/nu13113772.
7. Ahmad S, Zhao W, Renstrom F, Rasheed A, Zaidi M, Samuel M, et al. A novel interaction between the FLJ33534 locus and smoking in obesity: a genome-wide study of 14 131 Pakistani adults. Int J Obes (Lond). 2016;40(1):186-90. doi: 10.1038/ijo.2015.152.
8. Justice AE, Winkler TW, Feitosa MF, Graff M, Fisher VA, Young K, et al. Genome-wide meta-analysis of 241,258 adults accounting for smoking behaviour identifies novel loci for obesity traits. Nat Commun. 2017;8:14977. doi: 10.1038/ncomms14977.
9. Daily JW, Liu M, Park S. High genetic risk scores of SLIT3, PLEKHA5 and PPP2R2C variants increased insulin resistance and interacted with coffee and caffeine consumption in middle-aged adults. Nutr Metab Cardiovasc Dis. 2019;29(1):79-89. doi: 10.1016/j.numecd.2018.09.009.
10. Jin T, Youn J, Kim AN, Kang M, Kim K, Sung J, et al. Interactions of Habitual Coffee Consumption by Genetic Polymorphisms with the Risk of Prediabetes and Type 2 Diabetes Combined. Nutrients. 2020;12(8). doi: 10.3390/nu12082228.
11. Zheng JS, Arnett DK, Lee YC, Shen J, Parnell LD, Smith CE, et al. Genome-wide contribution of genotype by environment interaction to variation of diabetes-related traits. PLoS One. 2013;8(10):e77442. doi: 10.1371/journal.pone.0077442.
12. Kim J, Kim MK, Jung S, Lim JE, Shin MH, Kim YJ, et al. Interaction of iron status with single nucleotide polymorphisms on incidence of type 2 diabetes. PLoS One. 2017;12(4):e0175681. doi: 10.1371/journal.pone.0175681.
13. Franck M, de Toro-Martin J, Guenard F, Rudkowska I, Lemieux S, Lamarche B, et al. Prevention of Potential Adverse Metabolic Effects of a Supplementation with Omega-3 Fatty Acids Using a Genetic Score Approach. Lifestyle Genom. 2020;13(1):32-42. doi: 10.1159/000504022.
14. Simino J, Sung YJ, Kume R, Schwander K, Rao DC. Gene-alcohol interactions identify several novel blood pressure loci including a promising locus near SLC16A9. Front Genet. 2013;4:277. doi: 10.3389/fgene.2013.00277.
15. Feitosa MF, Kraja AT, Chasman DI, Sung YJ, Winkler TW, Ntalla I, et al. Novel genetic associations for blood pressure identified via gene-alcohol interaction in up to 570K individuals across multiple ancestries. PLoS One. 2018;13(6):e0198166. doi: 10.1371/journal.pone.0198166.
16. Kim Y, Kim J, Lim JE, Oh B, Won S, Kim MK. Genome-wide interaction study of single-nucleotide polymorphisms and alcohol consumption on blood pressure: The Ansan and Ansung

study of the Korean Genome and Epidemiology Study (KoGES). Genet Epidemiol. 2020;44(3):300-10. doi: 10.1002/gepi.22285.
17. Lin WY, Huang CC, Liu YL, Tsai SJ, Kuo PH. Genome-Wide Gene-Environment Interaction Analysis Using Set-Based Association Tests. Front Genet. 2018;9:715. doi: 10.3389/fgene.2018.00715.
18. Lin WY, Huang CC, Liu YL, Tsai SJ, Kuo PH. Polygenic approaches to detect gene-environment interactions when external information is unavailable. Brief Bioinform. 2019;20(6):2236-52. doi: 10.1093/bib/bby086.
19. He J, Kelly TN, Zhao Q, Li H, Huang J, Wang L, et al. Genome-wide association study identifies 8 novel loci associated with blood pressure responses to interventions in Han Chinese. Circ Cardiovasc Genet. 2013;6(6):598-607. doi: 10.1161/CIRCGENETICS.113.000307.
20. Osazuwa-Peters OL, Waken RJ, Schwander KL, Sung YJ, de Vries PS, Hartz SM, et al. Identifying blood pressure loci whose effects are modulated by multiple lifestyle exposures. Genet Epidemiol. 2020;44(6):629-41. doi: 10.1002/gepi.22292.
21. Sung YJ, de Las Fuentes L, Schwander KL, Simino J, Rao DC. Gene-smoking interactions identify several novel blood pressure loci in the Framingham Heart Study. Am J Hypertens. 2015;28(3):343-54. doi: 10.1093/ajh/hpu149.
22. Sung YJ, Winkler TW, de Las Fuentes L, Bentley AR, Brown MR, Kraja AT, et al. A Large-Scale Multi-ancestry Genome-wide Study Accounting for Smoking Behavior Identifies Multiple Significant Loci for Blood Pressure. Am J Hum Genet. 2018;102(3):375-400. doi: 10.1016/j.ajhg.2018.01.015.
23. Taylor JY, Schwander K, Kardia SL, Arnett D, Liang J, Hunt SC, et al. A Genome-wide study of blood pressure in African Americans accounting for gene-smoking interaction. Sci Rep. 2016;6:18812. doi: 10.1038/srep18812.
24. Hachiya T, Narita A, Ohmomo H, Sutoh Y, Komaki S, Tanno K, et al. Genome-wide analysis of polymorphism x sodium interaction effect on blood pressure identifies a novel 3'-BCL11B gene desert locus. Sci Rep. 2018;8(1):14162. doi: 10.1038/s41598-018-32074-1.
25. Li C, He J, Chen J, Zhao J, Gu D, Hixson JE, et al. Genome-Wide Gene-Potassium Interaction Analyses on Blood Pressure: The GenSalt Study (Genetic Epidemiology Network of Salt Sensitivity). Circ Cardiovasc Genet. 2017;10(6). doi: 10.1161/CIRCGENETICS.117.001811.
26. Li C, He J, Chen J, Zhao J, Gu D, Hixson JE, et al. Genome-Wide Gene-Sodium Interaction Analyses on Blood Pressure: The Genetic Epidemiology Network of Salt-Sensitivity Study. Hypertension. 2016;68(2):348-55. doi: 10.1161/HYPERTENSIONAHA.115.06765.
27. Park YM, Kwock CK, Kim K, Kim J, Yang YJ. Interaction between Single Nucleotide Polymorphism and Urinary Sodium, Potassium, and Sodium-Potassium Ratio on the Risk of Hypertension in Korean Adults. Nutrients. 2017;9(3). doi: 10.3390/nu9030235.
28. Jeong H, Jin HS, Kim SS, Shin D. Identifying Interactions between Dietary Sodium, Potassium, Sodium-Potassium Ratios, and FGF5 rs16998073 Variants and Their Associated Risk for Hypertension in Korean Adults. Nutrients. 2020;12(7). doi: 10.3390/nu12072121.
29. Francis M, Li C, Sun Y, Zhou J, Li X, Brenna JT, et al. Genome-wide association study of fish oil supplementation on lipid traits in 81,246 individuals reveals new gene-diet interaction loci. PLoS Genet. 2021;17(3):e1009431. doi: 10.1371/journal.pgen.1009431.
30. An P, Straka RJ, Pollin TI, Feitosa MF, Wojczynski MK, Daw EW, et al. Genome-wide association studies identified novel loci for non-high-density lipoprotein cholesterol and its postprandial lipemic response. Hum Genet. 2014;133(7):919-30. doi: 10.1007/s00439-014-1435-3.
31. Nishida Y, Hachiya T, Hara M, Shimanoe C, Tanaka K, Sutoh Y, et al. The interaction between ABCA1 polymorphism and physical activity on the HDL-cholesterol levels in a Japanese population. J Lipid Res. 2020;61(1):86-94. doi: 10.1194/jlr.P091546.
32. Kilpelainen TO, Bentley AR, Noordam R, Sung YJ, Schwander K, Winkler TW, et al. Multi-ancestry study of blood lipid levels identifies four loci interacting with physical activity. Nat Commun. 2019;10(1):376. doi: 10.1038/s41467-018-08008-w.

33. Liu M, Jin HS, Park S. Protein and fat intake interacts with the haplotype of PTPN11_rs11066325, RPH3A_rs886477, and OAS3_rs2072134 to modulate serum HDL concentrations in middle-aged people. Clin Nutr. 2020;39(3):942-9. doi: 10.1016/j.clnu.2019.03.039.
34. Noordam R, Bos MM, Wang H, Winkler TW, Bentley AR, Kilpelainen TO, et al. Multi-ancestry sleep-by-SNP interaction analysis in 126,926 individuals reveals lipid loci stratified by sleep duration. Nat Commun. 2019;10(1):5121. doi: 10.1038/s41467-019-12958-0.
35. Bentley AR, Sung YJ, Brown MR, Winkler TW, Kraja AT, Ntalla I, et al. Multi-ancestry genome-wide gene-smoking interaction study of 387,272 individuals identifies new loci associated with serum lipids. Nat Genet. 2019;51(4):636-48. doi: 10.1038/s41588-019-0378-y.
36. de Vries PS, Brown MR, Bentley AR, Sung YJ, Winkler TW, Ntalla I, et al. Multiancestry Genome-Wide Association Study of Lipid Levels Incorporating Gene-Alcohol Interactions. Am J Epidemiol. 2019;188(6):1033-54. doi: 10.1093/aje/kwz005.
37. Rudkowska I, Perusse L, Bellis C, Blangero J, Despres JP, Bouchard C, et al. Interaction between Common Genetic Variants and Total Fat Intake on Low-Density Lipoprotein Peak Particle Diameter: A Genome-Wide Association Study. J Nutrigenet Nutrigenomics. 2015;8(1):44-53. doi: 10.1159/000431151.
38. Tan A, Sun J, Xia N, Qin X, Hu Y, Zhang S, et al. A genome-wide association and gene-environment interaction study for serum triglycerides levels in a healthy Chinese male population. Hum Mol Genet. 2012;21(7):1658-64. doi: 10.1093/hmg/ddr587.
39. Wojczynski MK, Parnell LD, Pollin TI, Lai CQ, Feitosa MF, O'Connell JR, et al. Genome-wide association study of triglyceride response to a high-fat meal among participants of the NHLBI Genetics of Lipid Lowering Drugs and Diet Network (GOLDN). Metabolism. 2015;64(10):1359-71. doi: 10.1016/j.metabol.2015.07.001.
40. Sarzynski MA, Davidsen PK, Sung YJ, Hesselink MK, Schrauwen P, Rice TK, et al. Genomic and transcriptomic predictors of triglyceride response to regular exercise. Br J Sports Med. 2015;49(23):1524-31. doi: 10.1136/bjsports-2015-095179.
41. Coltell O, Sorli JV, Asensio EM, Barragan R, Gonzalez JI, Gimenez-Alba IM, et al. Genome-Wide Association Study for Serum Omega-3 and Omega-6 Polyunsaturated Fatty Acids: Exploratory Analysis of the Sex-Specific Effects and Dietary Modulation in Mediterranean Subjects with Metabolic Syndrome. Nutrients. 2020;12(2). doi: 10.3390/nu12020310.
42. Park S, Kang S. Alcohol, Carbohydrate, and Calcium Intakes and Smoking Interactions with APOA5 rs662799 and rs2266788 were Associated with Elevated Plasma Triglyceride Concentrations in a Cross-Sectional Study of Korean Adults. J Acad Nutr Diet. 2020;120(8):1318-29 e1. doi: 10.1016/j.jand.2020.01.009.
